# The Urinary Metabolome of Newborns with Perinatal Complications

**DOI:** 10.3390/metabo14010041

**Published:** 2024-01-10

**Authors:** Yamilé López-Hernández, Victoria Lima-Rogel, Rupasri Mandal, Jiamin Zheng, Lun Zhang, Eponine Oler, David Alejandro García-López, Claudia Torres-Calzada, Ana Ruth Mejía-Elizondo, Jenna Poelsner, Jesús Adrián López, Ashley Zubkowski, David S. Wishart

**Affiliations:** 1 Academic Unit of Biological Sciences, Metabolomics and Proteomics Laboratory, CONAHCyT-Autonomous University of Zacatecas, Zacatecas 98000, Mexico; 2 Hospital Central “Dr. Ignacio Morones Prieto”, San Luis Potosi 78290, Mexico; victoria.lima@uaslp.mx (V.L.-R.); ana.mejia@uaslp.mx (A.R.M.-E.); 3 The Metabolomics Innovation Centre, University of Alberta, Edmonton, AB T6G 1C9, Canada; rmandal@ualberta.ca (R.M.); jiamin3@ualberta.ca (J.Z.); lun2@ualberta.ca (L.Z.); azubkows@ualberta.ca (A.Z.); 4 Academic Unit of Biological Sciences, Autonomous University of Zacatecas, Zacatecas 98000, Mexico; davidrockerdagal@uaz.edu.mx; 5 Department of Biological Sciences, University of Alberta, Edmonton, AB T6G 1C9, Canada; ctorresc@ualberta.ca (C.T.-C.); poelzer@ualberta.ca (J.P.); 6 Academic Unit of Biological Sciences, microRNAs and Cancer Laboratory, Autonomous University of Zacatecas, Zacatecas 98000, Mexico; jalopez@uaz.edu.mx

**Keywords:** newborns, metabolites, metabolomics, LC-MS/MS, bronchopulmonary dysplasia, NICU, asphyxia

## Abstract

Maternal pathological conditions such as infections and chronic diseases, along with unexpected events during labor, can lead to life-threatening perinatal outcomes. These outcomes can have irreversible consequences throughout an individual’s entire life. Urinary metabolomics can provide valuable insights into early physiological adaptations in healthy newborns, as well as metabolic disturbances in premature infants or infants with birth complications. In the present study, we measured 180 metabolites and metabolite ratios in the urine of 13 healthy (hospital-discharged) and 38 critically ill newborns (admitted to the neonatal intensive care unit (NICU)). We used an in-house-developed targeted tandem mass spectrometry (MS/MS)-based metabolomic assay (TMIC Mega) combining liquid chromatography (LC-MS/MS) and flow injection analysis (FIA-MS/MS) to quantitatively analyze up to 26 classes of compounds. Average urinary concentrations (and ranges) for 167 different metabolites from 38 critically ill NICU newborns during their first 24 h of life were determined. Similar sets of urinary values were determined for the 13 healthy newborns. These reference data have been uploaded to the Human Metabolome Database. Urinary concentrations and ranges of 37 metabolites are reported for the first time for newborns. Significant differences were found in the urinary levels of 44 metabolites between healthy newborns and those admitted at the NICU. Metabolites such as acylcarnitines, amino acids and derivatives, biogenic amines, sugars, and organic acids are dysregulated in newborns with bronchopulmonary dysplasia (BPD), asphyxia, or newborns exposed to SARS-CoV-2 during the intrauterine period. Urine can serve as a valuable source of information for understanding metabolic alterations associated with life-threatening perinatal outcomes.

## 1. Introduction

According to the World Health Organization (WHO), 2.4 million newborns died in 2020 due to conditions and diseases related to a lack of quality care immediately after birth and during the first 28 days of life (the newborn period). Seventy-five percent of these deaths occurred during the first week of life. In 2019, about one million newborns died within 24 h of their birth [1].

Early neonatal outcomes greatly impact infant health and, in some cases, can have irreversible consequences for an individual’s entire life. Common perinatal complications often occur during labor or delivery due to various factors, such as antepartum hemorrhage, uterine rupture, abruptio placentae, premature rupture of membranes, malpresentation, shoulder dystocia, preterm birth, or post-term pregnancies, among others. Additionally, maternal pathological conditions, such as infections, can lead to neonatal pneumonia or early-onset sepsis. The presence of certain maternal diseases (i.e., gestational diabetes, preeclampsia, and eclampsia) and congenital malformations may also affect neonatal health [2]. These conditions can lead to oxygen deprivation or cerebral palsy, which are medical emergencies that require immediate attention in neonatal intensive care units (NICU). Exposure to hyperoxia can result in a release of free radicals and tissue damage due to oxidative stress and related diseases, such as preterm retinopathy (ROP), bronchopulmonary dysplasia (BPD), necrotizing enterocolitis (NEC), intraventricular hemorrhage (IVH), and hypoxic ischemic encephalopathy (HIE), among others. Lastly, the emergence of coronavirus disease 2019 (COVID-19) has raised concerns about vertical transmission in newborns from women who delivered while positive for SARS-CoV-2. Infections during pregnancy can be harmful to the fetus, possibly mediated by maternal immune activation and other inflammatory mechanisms. A recent cohort study of SARS-CoV-2 exposure in utero found preliminary evidence that maternal SARS-CoV-2 may be associated with neurodevelopmental sequelae in some offspring [3].

Bronchopulmonary dysplasia is one of the most commonly reported adverse outcomes in newborns, particularly in those who are born very prematurely (<30 weeks of gestation) and had birth weights less than 1500 g. BPD is a term used to define chronic lung disease in preterm infants. It often leads to higher rates of adverse neurological outcomes, including motor, visual, and auditory problems, lower average intelligence quotient (IQ), worse respiratory function, and more respiratory ill health compared to those of similar size and gestational age without BPD. This condition was first described in 1967 as a lung injury in preterm infants resulting from oxygen and mechanical ventilation [4]. Perinatal asphyxia is another serious condition that occurs when there is a lack of blood flow or gas exchange to/from the fetus immediately before, during, or after birth. This can be caused by maternal hemodynamic compromise, uterine rupture, abruptio placentae, and intrapartum infection (maternal fever in labor). Asphyxia often results in systemic and neurologic sequelae, respiratory distress, pulmonary hypertension, and liver, myocardial, and renal dysfunction.

During the first 24 h of life, neonatologists often face critical decisions regarding immediate interventions that could have important physiological consequences for the newborn. These interventions may include resuscitation procedures, mechanical ventilation, pharmacological treatments, or therapeutic hypothermia. Often, these actions must be taken quickly, and a considerable number of unexpected events or side effects could arise during emergency management. In this context, a comprehensive range of referential clinical and metabolic data are required to make more precise diagnoses and achieve better outcomes. Together with neonatal screening and routine blood tests, we believe that the enormous number of metabolites in urine can provide valuable insights about early physiological adaptations or stressors in very premature infants. Urine is one of the most used fluids in metabolomics and can be easily, abundantly, and safely collected in newborns [5].

In an effort to establish referential urinary metabolite values for newborn monitoring and screening, we recently characterized the urinary metabolome of healthy newborns [6]. Specifically, we compiled experimental metabolomics data using the TMIC Prime Assay and an updated literature review (until 2020) for absolute urinary metabolite concentrations detected in newborns without perinatal complications [6]. In the present study, we decided to extend this work and measure not only substantially more urinary metabolites but also establish quantitative referential urinary metabolome data for critically ill newborns. Specifically, we measured absolute concentrations for twice the previously reported number of urinary metabolites using a newly developed assay (the TMIC Mega Assay). This novel absolutely quantitative urinary assay was performed on the urine collected from both healthy newborns and newborns that were admitted to the NICU during 2021. This in-house assay includes up to 40 acylcarnitines, more than 60 compounds related to amino acid metabolism (amino acids, N-acetylated amino acids, biogenic amines, and amino acid derivatives), nearly 60 organic acids, microbial metabolites, short-chain fatty acids, and more than 30 lipid compounds, including sphingomyelins, lysophospholipids, and phosphatidylcholines. While all the NICU newborns survived, many developed chronic lung dysfunction (BPD). BPD typically arises as a consequence of life-saving postnatal interventions (such as oxygen delivery, mechanical ventilation, and corticosteroids), in combination with an aberrant reparative response to antenatal injury to the developing lungs caused by maternal conditions. We compared the urinary metabolomic profile from healthy newborns with NICU newborns, including those with BPD and perinatal asphyxia. Given the lack of literature reports for abnormal quantitative urinary values for most of the compounds evaluated here, these absolute concentrations could serve as valuable clinical reference values for neonatologists and clinical chemists. To make this information freely available, all the data were uploaded to the Human Metabolome Database (www.hmdb.ca).

## 2. Materials and Methods

### 2.1. Patient Recruitment

This cross-sectional study was conducted at the Hospital Central “Dr. Ignacio Morones Prieto” in San Luis Potosi, Mexico. The study received approval from the Research and Ethics Committee, with the registration 18–20 (CONBIOETICA-24-CEI-001-201604279), and adhered to the principles of the Declaration of Helsinki. Written informed consent was obtained from the parents of all subjects involved in the study. 

The study enrolled 38 critically ill neonates born between 30 April and 28 August 2021. The criteria for admission to the NICU are outlined in Table 1 (for mothers and newborns, respectively). The diagnosis of BPD was made based on current definitions established in 2001 by the National Institute of Child Health and Human Development (NICHD) workshop [7]. Infants born at or before 30 weeks gestational age who had been exposed to oxygen for 28 days were diagnosed with mild, moderate, or severe BPD at 36 weeks post-menstrual age based on their respiratory support at that time.

Separately, 13 healthy newborns (serving as reference controls) who were enrolled in a previous clinical research study (registration 18–20, CONBIOETICA-24-CEI-001-201604279) were included in this study.

### 2.2. Sample Collection

Blood samples were collected immediately after delivery for routine laboratory tests and the recommended neonatal screening (dried blood spot filters). Other parameters such as weight, size, cephalic perimeter, Apgar scores at 1 and 5 min, oxygen saturation (%), and respiratory and cardiac frequencies were also recorded. Maternal data were collected from the hospital records.

A urine sample was noninvasively collected from each newborn immediately after birth. The genital area was thoroughly cleaned, and a sterile bag was placed until micturition occurred. The urine sample in the sterile bag was then transferred via a micropipette to a sterile 1.5 mL Eppendorf tube, centrifuged at 3000 rpm to precipitate sediments, and stored in sterile microtubes at −80 °C until use. Additionally, urinary samples for the previously mentioned 13 healthy newborns were collected and stored in the same manner as described above.

### 2.3. Metabolite Measurement

A targeted absolutely quantitative LC-MS-based metabolomics assay was employed to analyze all the urine samples. This assay, called the TMIC Mega, uses a combination of direct injection (DI) mass spectrometry (MS) along with a reverse-phase LC-tandem mass spectrometry (MS/MS) run on an Applied Biosystems/MDS Analytical Technologies, Foster City, Canada) mass spectrometer. The Mega Assay can be used for the targeted identification and quantification of up to 250 different endogenous water-soluble metabolites, including amino acids and amino acid derivatives, biogenic amines, organic acids, nucleotide/nucleosides, acylcarnitines, glucose, and certain polar lipids.

Isotope-labeled internal standards (ISTDs) along with compounds covalently tagged with isotope-labeled chemical derivatization reagents are used for accurate metabolite quantification. The use of chemical derivatization also enhances ionization and improves reversed-phase LC separation. All chemicals used in this study were weighed individually on a Sartorius CPA225D semimicro electronic balance (Mississauga, ON, CA) with a precision of 0.0001 g. Stock solutions, with defined concentrations for each analyte, were prepared by dissolving the accurately weighed chemicals in proper solvents. Seven different calibration curve standards (Cal1 to Cal7) were prepared by mixing and diluting corresponding stock solutions with appropriate solvents, covering different concentration ranges for different analytes according to their known or expected normal/pathological concentrations in human urine. For analysis of amino acids, amino acid derivatives, biogenic amines, and nucleotide/nucleosides and organic acids, three quality control (QC) standards with different concentrations were prepared by diluting the Cal7 standard solution with the same solvents as used to prepare the calibration standards.

For amino acids, amino acid derivatives, biogenic amines, and nucleotide/nucleosides, OptimaTM-LC-MS-grade water (Fisher Scientific, Ottawa, ON, Canada) was used as the solvent for preparing stock solutions, calibration mixtures, QC mixtures, and a working ISTD solution mixture. The working ISTD solution mixture with defined concentrations of ISTDs in LC/MS-grade water was prepared by mixing all the isotope-labeled stock solutions. Chloroform (Sigma-Aldrich, Oakville, ON, Canada) was used for preparing stock solutions for lipids. Methanol was used for preparing acylcarnitines and hexose stock solutions. For lipids, acylcarnitines, and hexose, a working calibration mixture with defined concentrations of standards and a working ISTD solution mixture with defined concentrations of ISTDs in LC/MS-grade methanol were also made by mixing all the prepared stock solutions.

For organic acids, 75% (*v*/*v*) OptimaTM-LC-MS-grade methanol (Fisher Scientific, Ottawa, ON, Canada) in OptimaTM-LC-MS-grade water was used for preparing stock solutions and calibration mixtures. An ISTD solution mixture with standards having defined concentrations was prepared in the same way as the calibration standards. A working internal standard solution mixture was prepared by derivatizing the ISTD solution with an isotope-labeled chemical derivatization reagent (^13^C6-3-nitrophenylhydrazine or 3-NPH) during the sample preparation procedure. 

PITC derivatization was used to obtain the concentrations of amino acids, amino acid derivatives, and biogenic amines, and nucleotide/nucleosides. This panel uses a 96-deep-well plate (NuncTM 96 DeepWell plate, Fisher Scientific, Ottawa, ON, Canada) with a 96-well filter plate (Multiscreen “solvinert” filter plates, hydrophobic, PTFE, 0.45 μm, clear, nonsterile, Fisher Scientific, Ottawa, ON, Canada) attached via sealing tape, and a set of reagents and solvents used to prepare the plate assay. To each urine sample (or QC standard), the ISTD mixture solutions were pipetted directly onto the center of each corresponding spot/well in the upper filter plate. After drying the plate under a stream of nitrogen for 30 min, 50 μL of the 5% PITC derivatization solution (where 300 μL of PITC reagent (Sigma-Aldrich, Oakville, ON, Canada) was added to a mixture of ethanol (Fisher Scientific Ottawa, ON, Canada), LC/MS water, and pyridine (Sigma-Aldrich, Oakville, ON, Canada), each 1900 μL) was added to each well. The reaction was kept at room temperature for 20 min, followed by another 1.5 h drying under a gentle nitrogen stream to remove the excess PITC solution. To extract the targeted analytes, 300 μL of LC/MS-grade methanol containing 5 mM ammonium acetate (Fisher Scientific Ottawa, ON, Canada) was then added to each spot. The whole plate was covered and shaken at 300 rpm for 30 min at room temperature, and then centrifuged at 50× *g* for 5 min to collect the extracts from the upper filter plate to the bottom collection plate. Finally, 50 μL of extracts were transferred to a new 96-deep-well plate and then diluted with 450 μL of LC/MS-grade water for LC-MS/MS analysis to quantify amino acids, amino acid derivatives, biogenic amines, and nucleotide/nucleosides. Moreover, 10 μL of the remaining extracts were transferred to another new 96-deep-well plate and then diluted with 490 μL of DFI buffer for direct flow injection-tandem mass spectrometry (DFI-MS/MS) analysis. This was conducted to quantify the lipids, acylcarnitines, and glucose/hexose (which are not derivatized by PITC). The assay was completed to quantify the lipids, acylcarnitines, and glucose/hexose.

For organic acid analysis of the urine samples, 50 µL of each sample was directly loaded into a 96-well plate. Then, 75 µL of derivatization reagent (which consisted of 25 µL of 250 mM 3-NPH (Sigma-Aldrich, Oakville, ON, Canada) in 50% aqueous methanol, 25 µL of 150 mM 1-ethyl-3-(3-(dimethylamino)propyl) carbodiimide (EDC, Sigma-Aldrich, Oakville, ON, Canada) in methanol, and 25 µL of 7.5% pyridine in 75% aqueous methanol) was added to each well. In a 1.5-mL Eppendorf tube, 125 µL of the working ISTD solution was prepared by mixing 50 µL of the ISTD mixture solution with 75 µL of isotope-labelled derivatization reagent (which consisted of 25 µL of 250 mM ^13^C6-3-NPH (Cayman chemical, Ann Arbor, MI, USA) in 50% aqueous methanol, 25 µL of 150 mM 1-ethyl-3-(3-(dimethylamino)propyl) carbodiimide (EDC) in methanol, and 25 µL of 7.5% pyridine in 75% aqueous methanol). The 96-well plate was then shaken at 500 rpm for 2 h at room temperature, followed by adding 325 µL of LC/MS water and 50 µL of butylated hydroxytoluene (BHT) dissolved in methanol (2 mg/mL) to each well of the plate. The working ISTD solution was shaken along with the plate under the same conditions for 2 h and then diluted with 1125 µL of LC/MS water. Moreover, 10 µL of the diluted working ISTD solution was then loaded to each well of a new deep well plate except for the double blank sample position, followed by transferring 25 µL of the derivatized samples to the corresponding wells of the new plate. 

#### LC/DFI-MS/MS Analysis

Mass spectrometric analysis was performed on an ABSciex 5500 QTrap^®^ tandem mass (MS/MS) spectrometer (Applied Biosystems/MDS Analytical Technologies, Foster City, CA, USA) equipped with an Agilent 1290 series UHPLC system (Agilent Technologies, Palo Alto, CA, USA). An Agilent reversed-phase Zorbax Eclipse XDB C18 column (3.0 mm × 100 mm, 3.5 μm particle size, 80 Å pore size) with a Phenomenex (Torrance, CA, USA) A SecurityGuard C18 guard column (4.0 mm × 3.0 mm) was used for LC-MS/MS analysis. The controlling software for the sample analysis was Analyst 1.7.2 (Applied Biosystems/MDS Analytical Technologies, Foster City, CA, USA). Data analysis was completed using MultiQuantTM 3.0.3 (Applied Biosystems/MDS Analytical Technologies, Foster City, CA, USA).

The HPLC parameters used for the LC-MS/MS analysis of the PITC panel were as follows: solvent A: 0.2% (*v*/*v*) formic acid in water, and solvent B: 0.2% (*v*/*v*) formic acid in acetonitrile. The gradient profile for this UHPLC solvent run was as follows: t = 0 min, 0% B; t = 0.5 min, 0% B; t = 5.5 min, 95% B; t = 6.5 min, 95% B; t = 7.0 min, 0% B; and t = 9.5 min, 0% B. The column oven was set at 50 °C. The flow rate was 500 μL/min, and the sample injection volume was 10 μL. The mass spectrometer was set to a positive electrospray ionization mode with a scheduled multiple reaction monitoring (MRM) scan. The IonSpray voltage was set at 5500 V and the temperature at 500 °C. The curtain gas (CUR), ion source gas 1 (GAS1), ion source gas 2 (GAS2), and collision gas (CAD) were set at 20, 40, 50, and medium, respectively. The entrance potential (EP) was set to 15 V. The declustering potential (DP), collision energy (CE), collision cell exit potential (CXP), MRM precursor ion (Q1), and fragment ion (Q3) were optimized and set individually for each analyte and isotope-labeled ISTD.

For DFI-MS/MS analysis, the UHPLC autosampler was connected directly to the MS ion source via red PEEK tubing. The DFI buffer mentioned above was used as the mobile phase. The flow rate was programmed as follows: t = 0 min, 30 μL/min; t = 1.6 min, 30 μL/min; t = 2.4 min; 200 μL/min; t = 2.8 min, 200 μL/min; and t = 3.0 min, 30 μL/min. The sample injection volume was 20 μL. The IonSpray voltage was set at 5500 V and the temperature was set at 200 °C. The CUR, GAS1, GAS2, and CAD were set at 20, 40, 50, and medium, respectively. The EP and CXP were set at 10 and 15 V, respectively, for positive mode and −10 and −15 V, respectively, for negative mode. Likewise, the DP, CE, Q1, and Q3 were optimized and set individually for each analyte and ISTD.

For the separation of organic acids by LC-MS/MS, the HPLC solvents used were (A) 0.01% (*v*/*v*) formic acid in water and (B) 0.01% (*v*/*v*) formic acid in acetonitrile. The gradient profile was as follows: t = 0 min, 25% B; t = 6.0 min, 65% B; t = 6.3 min, 90% B; t = 6.5 min, 100% B; t = 7.0 min, 100% B; t = 7.5 min, 25% B; t = 12.0 min, 25% B. The column oven was set to 40 °C. The flow rate was 400 μL/min, and the sample injection volume was 10 μL. The mass spectrometer was set to a negative electrospray ionization mode with scheduled MRM scanning. The IonSpray voltage was set at −4500 V and the temperature at 400 °C. The CUR, GAS1, GAS2, and CAD were set at 20, 30, 30, and medium separately. The EP was set at −10 V, and the DP, CE, CXP, Q1, and Q3 were optimized and set individually for all the analytes and isotope-labeled ISTDs.

### 2.4. Statistical Analysis

Maternal characteristics and perinatal outcomes were described using medians with interquartile ranges (IQRs) or means (with standard deviations (s.d.)) and frequencies (%) for continuous and categorical data, respectively. The D’Agostino–Pearson normality test was used to assess normality. The Student’s *t*-test or the Mann–Whitney test were used for assessing the significance of continuous data, while Pearson Chi^2^ tests or Fisher’s exact tests were used for assessing the significance of categorical variables. A *p*-value less than 0.05 (*p*  <  0.05) was considered statistically significant. Analyses were conducted using SPSS (IBM, version 24).

Metabolite analysis was performed with MetaboAnalyst 5.0 [8]. Metabolites with more than 25% of missing values were excluded from further analysis. For the remaining metabolites, values below the limit of detection (LOD) were imputed using 1/5 of the minimum positive value of each variable. The data were then normalized using various strategies (creatinine normalization, quantile normalization, normalization based on the total sum of organic acids, and total sum of metabolites). For subsequent analysis, metabolite values were expressed as micromol/mmol creatinine, and quantile normalization, log transformation, and autoscaling were selected to generate appropriate Gaussian metabolite concentration distributions. Differences in mean metabolite concentration values between different conditions were assessed using a parametric *t*-test or one-way ANOVA (adjusted *p*-value (FDR) cut-off = 0.05). Principal component analysis (PCA) and two-dimensional partial least squares discriminant analysis (2-D PLS-DA) scores plots were used to compare metabolite data between study groups. Permutation tests (2000-fold) were used to assess statistical significance and minimize the possibility that the observed separation of the PLS-DA clusters was due to chance. Differentiated metabolites were identified by a variable importance in projection (VIP) using a score cutoff of  >1.5. Heatmaps of the top 25 significant metabolites (via *t*-test) were created via MetaboAnalyst. 

## 3. Results

### 3.1. Healthy and NICU Newborns: Clinical and Metabolic Characteristics

The perinatal characteristics of the newborns in the NICU compared to healthy newborns are shown in Table 2. Gestational age, APGAR at 1 and 5 min, weight, length, and cephalic perimeter were significantly lower in the NICU newborns (*p* < 0.05). Cardiac and respiratory rates and inspirated frequency were higher in the NICU newborns (*p* < 0.05). Routine clinical laboratory tests were not conducted in the healthy newborns. Asphyxiated newborns had pH values ranging from 6.9 to 7.22; H_2_CO_3_: 6.7–23; blood lactate 3–15 mmoL/L, and an APGAR range: 1–7 at five minutes. All of them were monitored with an amplitude-integrated encephalogram (aEEG). Newborns with APGAR 8 at five minutes were non-asphyxiated, and they were assisted with non-invasive ventilation (nCPAP). A Fenton chart was used as a growth reference, classifying a newborn as small for gestational age/IUGR if the weight was lower than the 10th centile [9].

Absolute urinary concentrations of 268 metabolites were measured by LC-MS/MS and FIA-MS/MS in NICU newborns. After removing metabolites that were below the limit of detection (LOD) in >25% of samples, 167 metabolites were left for further analysis. Supplementary Table S1 shows the total number of metabolites measured. For classification into compound classes, ClassyFire (http://classyfire.wishartlab.com, accessed on 20 November 2023) was used. ClassyFire is a web-based application for automated structural classification of chemical entities. This application uses a rule-based approach that relies on a comprehensive and computable chemical taxonomy [10].

Supplementary Table S2 shows the urinary concentrations measured in this study (expressed as median (IQR) or mean +/− s.d.) for NICU newborns, along with their normal and abnormal urinary concentrations (deposited at version 5.0 (www.hmdb.ca). An additional search was performed in Pubmed with the search terms: “metabolite name” AND urin* AND concentration AND (newborn* OR neonate* OR infant*) AND (NICU or “neonatal intensive care unit, accessed on 30 May 2023). The concentration values (when available) were included in the Supplementary Table S2 [6,11,12,13,14,15,16,17,18,19,20,21,22,23,24,25,26,27,28,29,30,31,32,33,34,35,36,37,38,39,40,41,42,43,44,45,46,47,48,49,50,51,52]. Notably for 37 metabolites measured in this study, there were no existing literature reports on urinary concentration levels (quantitative results) in newborns or infants under the selected criteria.

### 3.2. Clinical Characteristics, Perinatal Outcomes, and Maternal History of Newborns with Bronchopulmonary Dysplasia (BPD)

The perinatal outcomes of newborns diagnosed with BPD (*n* = 12) compared to those without a BPD diagnosis are shown in Supplementary Table S3. The maternal history for both groups is also included. The newborns diagnosed with BPD had significantly lower gestational age (*p* < 0.0001), weight (*p* = 0.0005), size (*p* = 0.0003), and cephalic perimeter (*p* = 0.001). On average, the newborns with BPD spent more days in the NICU (*p* < 0.0001), with nine out of twelve requiring intubation. Furthermore, 11 newborns were exposed to oxygen for more than one month, and all 12 of them were discharged with oxygen supplementation. Respiratory distress, intrauterine pneumonia, and patent ductus arteriosus were more frequent in BPD newborns. The levels of leukocytes, neutrophils, and creatinine were also significantly lower (*p* < 0.05) in BPD newborns. Likewise, the presence of IgG antibody titer against SARS-CoV2 was significantly higher in BPD newborns. Regarding maternal antecedents, only premature labor and prenatal steroids were significantly higher in the BPD group.

### 3.3. Comparison between Healthy and NICU Newborns

**Figure 1:**
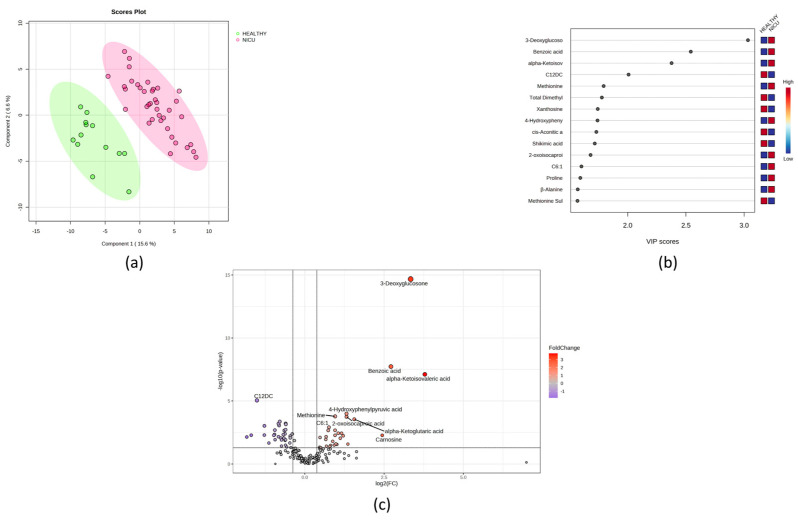
Univariate and multivariate analysis of the NICU newborn urine samples compared to those of heathy controls. (**a**) PLS-DA score plot comparing healthy newborn urine (green) with NICU newborn urine (red). (**b**) Rank of the different metabolites (the top 15) identified by the PLS-DA according to the VIP score on the *x*-axis. The most discriminating metabolites are shown in descending order of their coefficient scores. The colored boxes indicate whether metabolite concentration is increased (red) or decreased (blue) in NICU newborns relative to healthy newborns. (**c**) Volcano plot showing upregulated metabolites (red) and downregulated metabolites (blue) in NICU newborns compared to healthy controls.

A summary of the results of the univariate and multivariate analyses for this study is shown in Figure 1. The fold change analysis and VIP score plot revealed that 3-deoxyglucosone, benzoic acid, and alpha keto-isovaleric acid were significantly increased in the urine of NICU newborns. The univariate analysis also revealed that 44 metabolites were significantly dysregulated in NICU newborns after correcting for multiple comparisons (FDR < 0.05). Supplementary Table S4 shows the fold change (FC) values for the significant metabolites. The global enrichment test (relative betweenness centrality) revealed that multiple metabolic pathways related to amino acid metabolism and mitochondrial processes are dysregulated (Supplementary Table S5).

After adjusting for confounders (gestational age, resolution, and weight), 3-deoxyglucosone remained significant in differentiating newborns admitted to the NICU from those who were not (healthy newborns).

### 3.4. Urinary Metabolites Altered in BPD and Asphyxiated Newborns

**Figure 2:**
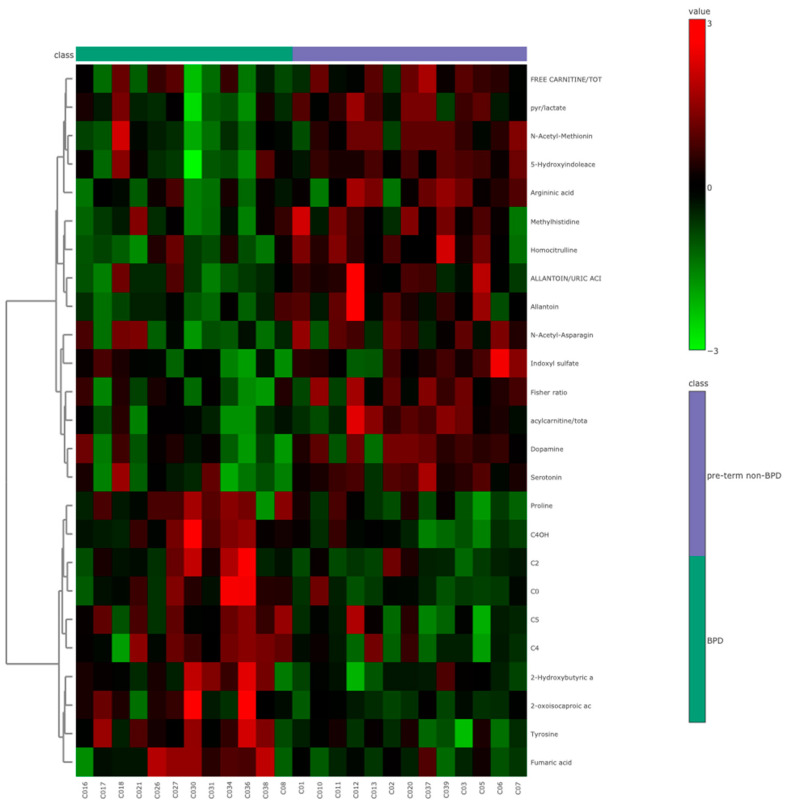
A representative heatmap of the top 25 significant metabolites (*t*-test) comparing BPD and non-BPD newborns.

Of the 26 newborns without a BPD diagnosis, 13 were premature (non-BPD group) and 13 were full-term newborns. Among them, eight experienced asphyxia during delivery (asphyxia group). A comparison was then conducted between preterm newborns with and without a BPD diagnosis. The volcano plot analysis showed that 13 metabolites were significantly upregulated, while 15 were downregulated (*p* < 0.05) among preterm newborns with BPD. None of the metabolites remained significant after multiple comparison test correction (FDR < 0.05). Among the measured clinical values, oxygen exposure time, intubation, respiratory distress syndrome, discharge with oxygen, and low weight at birth were the most relevant variables distinguishing BPD from non-BPD newborns. Some organic acids (fumaric acid, 2-oxoisocaproic acid, and 2-hydroxybutyric acid), amino acids (proline and tyrosine), and acylcarnitines (C0, C2, C4OH, C4, C5, and C5:1DC) were higher in the BPD group. Serotonin, 5-hydroxyl indoleacetic acid, indoxyl sulfate, and other amino acid derivatives (allantoin and homocitrulline) were found in lower levels (Figure 2).

Although resolution mode was not significantly associated with BPD outcome (*p* > 0.05) in our study, we evaluated how resolution mode could influence the urinary metabolic profile of preterm newborns who developed BPD. We found 20 metabolites dysregulated based on the volcano plot analysis. Dysregulated metabolites were amino acids and derivatives, biogenic amines, and organic acids. Of note, two microbial metabolites (2,5-Furandicarboxylic acid and indolelactic acid) were found downregulated in BPD newborns born through cesarean section, and 3-methoxytyramine, related to aromatic amino acids (phenylalanine and tyrosine) metabolism by gut microbiota, was found altered (Supplementary Figure S1).

**Figure 3:**
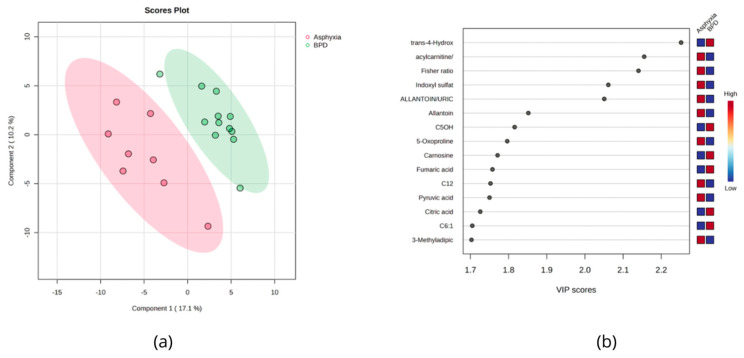
(**a**) PLS-DA score plot comparing BPD newborn urine (green) with asphyxiated newborn urine (red). (**b**) Rank of the different metabolites (the top 15) identified by the PLS-DA according to the VIP score on the *x*-axis. The most discriminating metabolites are shown in descending order of their coefficient scores. The colored boxes indicate whether metabolite concentration is increased (red) or decreased (blue).

The urine metabolome data of BPD newborns were also compared with those who were asphyxiated during labor. A total of 29 metabolites were found to be significantly different (*p* < 0.05). The volcano plots show that 15 metabolites were downregulated in the asphyxia group, while 14 were found upregulated (FC = 1.3). Urinary trans-hydroxyproline was found to be increased in BPD newborns (*p* = 5 × 10^−4^), while indoxyl sulfate (*p* = 2 × 10^−3^), allantoin (*p* = 7 × 10^−3^), the acetylcarnitine/total carnitine ratio (10^−3^), the Fisher ratio (10^−3^), and the allantoin/uric acid ratio (2 × 10^−3^) were found to be higher in the asphyxiated group (Figure 3).

### 3.5. NICU Newborns Exposed to SARS-CoV-2 during Pregnancy

In this study, none of the newborns tested positive for SARS-CoV-2 as per a standard PCR test. However, nine newborns had IgG antibody titers, while one had IgM titers indicating SARS-CoV-2 exposure. Among these ten newborns, six developed BPD, three were preterm newborns without BPD, and one was an asphyxiated baby. None of the mothers were vaccinated at the time of the sample collection. 

Nine metabolites were found to be statistically different in the urine of SARS-CoV-2-exposed newborns. Glucose (*p* = 0.0003) was found in higher levels in IgG/IgM-positive newborns. Conversely, nudifloramide (*p* = 0.003), carnitine (*p* = 0.01), allantoin (*p* = 0.01), glutamine (*p* = 0.01), methylhistidine (*p* = 0.01), 3-methyladipic acid (*p* = 0.01), quinolinic acid (*p* = 0.04), and betaine (*p* = 0.04) were found in lower levels. 

## 4. Discussion

Neonatal metabolomics can offer invaluable and comprehensive data about a newborn’s clinical status, providing important insights into the complex metabolic changes that occur during adaptation to extrauterine life. Among the biofluids that can be collected from newborns, urine is particularly appealing due to its availability, abundance, and the fact that it can be collected noninvasively. Given that approximately 4500 metabolites can be detected and/or quantified in human urine, this chemical richness makes it an excellent source of information about physiological and pathological processes [53]. 

Our group previously reported on the urinary metabolome of healthy newborns [6]. In that earlier work, only full-term healthy neonates born in maternal hospital rooms were included. That previous study quantified 136 metabolites in urine collected within the first 24 h of life, with 86 of them being reported for the first time in neonates. With the constant advancement of metabolomics technologies, it is now possible to measure absolute concentrations of many more metabolites in a wide variety of biofluids—especially for adults. However, a simple search through metabolomics databases and the existing literature indicates that there continues to be a significant lack of quantitative data for urinary metabolites, not only for healthy newborns but also for unhealthy ones as well. In the present work, using the newly developed TMIC Mega Assay, we were able to increase the number of quantified urinary metabolites in newborns from 136 to 268. After data cleaning and filtering, a final set of 180 quantified metabolites and ratios were calculated and uploaded to the Human Metabolome Database (https://www.hmdb.ca). Of the 180 metabolites and metabolite ratios determined for this study, 37 were reported for the first time in newborns. 

When urinary metabolome data for healthy newborns were compared with newborns admitted to NICU, differences were found for the levels of 44 metabolites. Despite urine samples being collected during the first 24 h of life, the effect of different nutritional patterns and treatments/interventions carried out during this period cannot be disregarded. Healthy newborns were breastfed, whereas NICU newborns were immediately given intravenous fluids, antibiotics (28 out of 38), and other treatments. Newborns who experienced asphyxiation during labor underwent therapeutic hypothermia (seven out of thirty-eight), while extremely premature newborns also received surfactants (five out of thirty-eight), caffeine (seven out of thirty-eight), and nitric oxide (one out of thirty-eight) as part of their treatment. These interventions should be taken into account when interpretating the results due to their potential influence on the metabolic profiles of the newborns, as discussed below.

One of the more remarkable findings of this study included the elevated levels of urinary 3-Deoxyglucosone (3DG) found in the NICU newborns. Indeed, 3DG is a highly reactive species, and its accumulation induces oxidative stress (OS). Moreover, 3DG is widely recognized as a precursor to advanced glycation end products (AGEs), which are formed by the nonenzymatic reaction of glucose and other glycating compounds with proteins. In preterm newborns, OS results from an imbalance between oxidant and antioxidant systems, leading to oxidative damage to multiple organs and systems. OS is a key factor in the development of several conditions, such as BPD, necrotizing enterocolitis, intraventricular hemorrhage, kidney damage, respiratory distress syndrome, and patent ductus arteriosus [54]. These reactive intermediates are likely generated during standard intensive care procedures, such as reanimation, intubation, parenteral nutrition, medications, etc. It is worth mentioning that 3DG can also be produced by sterilizing glucose solutions with heat. Haybrard et al. [55] demonstrated that the initial amount of glucose, supplier, mean oxygen permeability coefficient, type of container materials, and storage duration since manufacture could influence the formation of glucose degradation products. So, it is possible that 3DG may, in some cases, be introduced exogenously through intravenous feeding via sterilized glucose solutions. Regardless of how 3DG accumulates (whether produced endogenously or acquired exogenously), once in circulation, both nervous system and organ damage could occur. 

Benzoic acid is another urinary metabolite that was found to be elevated in NICU newborns relative to healthy newborns. Benzoic acid may have increased for at least two reasons. One possibility is that it is being introduced exogenously. Benzoic acid is commonly used as a preservative for many intravenous solutions used in the NICU, and neonates seem to lack the capacity to conjugate benzoic acid with glycine (which produces harmless hippuric acid). It is also worth noting that a build-up of benzoic acid can cause metabolic acidosis and neurotoxicity [56]. A second possibility is that benzoic acid may be arising endogenously. Benzoic acid is a well-known product of gut microbial catabolism of phenylalanine and tyrosine. Along with hippuric acid (which was also found to be elevated in NICU newborns), benzoic acid constitutes a gut microbial marker, produced mainly by *Escherichia coli*, *Bifidobacterium lactis*, *Lactobacillus gasseri*, and *Collinsella*, among others. In this regard, our data on benzoic acid suggest that NICU newborns may either have a different gut microbiota composition than healthy newborns or that the resident microbiota in NICU newborns are handling a greater flux of aromatic amino acids than healthy newborns.

Newborns diagnosed with BPD showed increased levels of urinary tyrosine. Transient neonatal tyrosinemia (TNT), a common amino acid metabolism disorder in premature newborns [57], is known to cause this increase in urinary tyrosine. TNT can also result from an intake of phenylalanine and tyrosine during parenteral nutrition in 90% of premature newborns [58]. An overload of the catabolic pathway of phenylalanine towards oxidation could be another possible cause of increased levels of tyrosine as proteins are targets for reactive oxygen species (ROS) generated under OS. During the neonatal period, one of the main reactions taking place as a result of ROS is the oxidation of phenylalanine (Phe) to ortho-tyrosine (o-Tyr) or meta-tyrosine (m-Tyr) [59]. Another useful biomarker of nitrosative stress is 3-nitrotyrosine (3NO_2_-Tyr). However, all the samples showed values under the LOD for this metabolite, leading to its exclusion from further analysis. A significant increase in proline was found in BPD newborns. Proline plays a major role in arginine synthesis in human preterm infants [60] essential for ammonia disposal via urea synthesis and is associated with collagen synthesis for lung repair [61]. Organic acids and acylcarnitines were also found to be increased in BPD newborns, probably due to dysregulated energy metabolism and mitochondrial dysfunction in these very preterm infants.

It is worth noting that the levels of two closely related metabolites, serotonin and 5-hydroxyindoleacetic acid, were found to be decreased in the urine of BPD newborns. Moreover, 5-hydroxyindoleacetic acid (5-HIAA) is the primary metabolite of serotonin and plays a crucial role in fetal and postnatal brain development [62]. Premature newborns exposed to hypoxic–ischemic insult often develop brain damage. Buller et al. [62] hypothesized that the serotonergic network in the brain is significantly disrupted following a preterm hypoxic–ischemic injury. However, there are no reports of alterations in serotonin and 5-hydroxyindoleacetic acid levels associated with BPD. Converging evidence suggests that preterm newborns show profound changes in serotonin transporter gene transcription, with some studies suggesting that these alterations can be specifically attributed to postnatal stress [63]. Moreover, serotonin is a key metabolite involved in the synthesis of melatonin, which is neuroprotective to the fetal brain due to its antiapoptotic, antioxidant, and anti-inflammatory effects [64].

Some microbial metabolites were found dysregulated in BPD newborns when the resolution mode (vaginal or cesarean section) was compared. Moreover, 2,5-Furandicarboxylic acid is a microbial metabolite, a product of the oxidation of hydroxymethylfurfural (HMF) by the enzyme furfural/HMF oxidoreductase, which is found in the Gram-negative environmental bacterium *Cupriavidus basilensis* (family Burkholderiaceae). Dissemination of these environmental pathogens in hospital plumbing systems has been reported, driving nosocomial infection [65], and clinical isolates have been collected and sequenced. Moreover, 2,5-Furandicarboxylic acid increases with the level of fructose consumed [66].

Indolelactic acid is also a microbial metabolite; urinary indole-3-lactate is produced by Clostridium sporogenes [67]. Indole formation from tryptophan occurs through the activation of the enzyme tryptophanase (TnaA), which can be found in many Gram-negative and Gram-positive bacterial species, including *Escherichia coli*, *Clostridium* sp., and *Bacteroides* sp. [68,69]. It is also produced by *Firmicutes*, *Actinobacteria,* and *Proteobacteria* phylum [70]. Interestingly, another metabolite related to aromatic amino acids (phenylalanine and tyrosine) was found altered. Furthermore, 3-methoxytyramine is a dopamine metabolite and is considered a neurotransmitter and a neuromodulator that in certain situations may be involved in movement control and abnormal dopaminergic transmission [71].

Allantoin, an oxidation product of uric acid and an “in vivo” marker of free radical generation, was found to be decreased in caffeine-treated BPD newborns. Oxidized ascorbic acid, uric acid, allantoin, and *o*-tyrosine have been previously found in tracheal lavage fluid and urine during the first days of life in infants who later developed BPD [72]. In our study, treatment with caffeine in the BPD group likely reduced the effects of oxidative stress and oxidative stress biomarkers such as allantoin, as has been previously demonstrated in a clinical trial [73]. This study showed that the incidence of BPD in infants who weighed <1250 g at birth was reduced when caffeine was used to treat these infants, improving survival and neurodevelopment through 11 years of follow-up. Antioxidant, anti-inflammatory, and antiapoptotic properties of caffeine and its ability to scavenge reactive oxygen species may contribute to its lung- and neuro-protective effects in premature infants. 

When the urinary data of BPD newborns were compared with asphyxiated newborns, allantoin and the allantoin/uric acid ratio were found elevated in the latter group. It is worth mentioning that asphyxiated newborns were subject to therapeutic hypothermia immediately after delivery. The goal of therapeutic hypothermia is to minimize damage from secondary neuronal injury, decreasing mortality and severe disability. However, side effects including peripheral vasoconstriction, diuresis, cardiac dysfunction, arrhythmias, coagulopathy, thrombocytopenia, leukocyte dysfunction, and pulmonary hypertension must be carefully monitored [74]. From a molecular perspective, during asphyxia, the interruption of oxygen and glucose supply to the brain leads to a decrease in ATP and a failure of the ATP-dependent Na/K pump. This triggers an influx of sodium and water into the cell, resulting in cellular lysis and the release of glutamate. The subsequent increase in intracellular calcium initiates oxidative stress reactions, a shift to anaerobic metabolism, metabolic acidosis, and failure of mitochondrial activity, ultimately leading to irreversible cell death [75]. TCA cycle intermediates (citrate, α-ketoglutarate, cis-aconitate, and succinate), pyruvate, DMA, DMG, lactose, and galactose have been found to be increased in urine in HIE newborns during the first month of life [76,77]. Sarafidis et al. [78] reported higher urinary pyruvate levels in newborns with HIE (treated and nontreated with therapeutic hypothermia) compared to controls at 3 days of life. Succinate levels have been found in lower levels in urine from asphyxiated patients (with or without HIE) in comparison to healthy controls [79]. In our study, Indoxyl sulfate, a uremic toxin, was also found to be elevated in asphyxiated subjects. Indoxyl sulfate is a product of indole metabolism that is produced from tryptophan by intestinal microbiota such as *Escherichia coli* and is frequently associated with kidney dysfunction [80]. Also, in our study, BPD newborns had elevated levels of trans-hydroxyproline, proline, and N-acetyl proline in comparison with asphyxiated newborns, revealing a mechanism that seems to be associated with lung repair [81].

Our results show metabolites that have been consistently linked with SARS-CoV-2 infections in different studies. Elevated glucose levels were found in newborns with IgG titers, along with levels of carnitine and acylcarnitines, while glutamine was found in lower concentrations. This is consistent with most of the current findings in patients that have active SARS-CoV-2 infections. SARS-CoV-2 infection has been associated with a higher risk of prematurity [82], premature rupture of membranes at term, and neonatal intensive care unit admissions [83]. To our knowledge, this is the first study evaluating urinary metabolites in newborns exposed to SARS-CoV-2 during intrauterine life. A few metabolomics studies have been conducted that examined cord blood [84] and plasma [85]. A study involving cord blood plasma samples from 23 mild COVID-19 cases (mother infected/newborn negative) and 23 gestational age-matched controls found significant differences in the concentration of 19 metabolites between the groups (*p*-value < 0.05). Elevated levels of glucocorticoids, pyruvate, lactate, purine metabolites, phenylalanine, and branched-chain amino acids of valine and isoleucine were found in cases, while ceramide subclasses were decreased [84]. In the second SARS-CoV-2 metabolomics study conducted on plasma involving 20 neonates (10 cases and 10 controls), the authors suggested that neonates born to SARS-CoV-2-positive mothers, without evidence of viral infection at birth, have a distinct plasma lipidomic and metabolomic profile compared to those of uninfected mothers [85]. However, none of these previous studies evaluated how this factor could contribute to negative outcomes for the newborn.

## 5. Conclusions

This study provides quantitative data for more than 160 urinary metabolites measured (some of them for the first time) during the first 24 h in newborns admitted to the NICU. Our metabolomic data suggest that oxidative stress is highly prevalent among NICU infants and that it may be one of the principal causes of organ or neural damage suffered by newborns as a result of antenatal or perinatal events (such as perinatal asphyxia, bronchopulmonary dysplasia, or SARS-CoV2 exposure). This comprehensive analysis provides a better understanding of the pathophysiologic processes occurring in preterm infants. The diagnosis of BPD is not the end of the road; this diagnosis marks a functional pulmonary impairment with potential lifelong consequences for the infant/child/adult. To the extent that more predictors such as risk factors or therapeutic targets can be added, the clinical management of this disease will greatly improve. In an effort to provide useful reference data to the neonatal care community and to help with the monitoring of early infant health outcomes, we have uploaded all the NICU and healthy newborn metabolite data to the HMDB. 

## Figures and Tables

**Table 1:** Maternal conditions for NICU admission.

Maternal Conditions	*N* (%)	Neonatal Conditions	*N* (%)
Preeclampsia	5 (13)	Transient Tachypnea	17 (44.7)
Gestational Diabetes	6 (15.8)	Intrauterine Pneumonia	17 (44.7)
Obesity	1 (2.6)	Persistent Pulmonary Hypertension	6 (15.8)
Drug Abuse	3 (7.9)	Pneumothorax	2 (5.3)
AIDS	1 (2.6)	Respiratory Hypoxemic Failure	1 (2.6)
Anemia	2 (5.3)	Early Onset Sepsis	8 (21.1)
Urinary Infection	13 (34.2)	Necrotizing Enterocolitis 1 A	2 (5.3)
Premature Labor	26 (68.4)	Meconium Aspiration Syndrome	3 (7.9)
Placenta Previa	4 (10.5)	Neonatal Depression by Anesthetics	3 (7.9)
Loss of Fetal Reactivity	8 (21.1)	Hypoxic Ischemic Encephalopathy stage II with seizures	3 (7.9)
Chorioamnionitis	1 (2.6)	Hypoxic Ischemic Encephalopathy stage II without seizures	6 (15.8)
Premature Rupture of Membranes	4 (10.5)	Intrauterine Growth Restriction	3 (7.9)
Multiple Pregnancies	9 (23.7)	Liver Hematoma	2 (5.3)
Abruptio Placentae	1 (2.6)	Patent Ductus Arteriosus	5 (13.2)
Achondroplasya	1 (2.6)	Distress Respiratory Syndrome	6 (15.8)
Cervicitis	6 (15.8)		
Obstetric Trauma	1 (2.6)		
Asthma	1 (2.6)		

**Table 2:** Perinatal characteristics of NICU and healthy newborns.

Variable	Healthy (*N* = 13)	NICU (*N* = 38)	*p*-Value *
Male Gender, n (%)	7 (63.6)	29 (76.3)	0.4016
Vaginal Birth, n (%)	3 (27.3)	20 (52.6)	0.1805
Gestational Age (wks)	38.1 (1.2)	35.6 (3)	**0.007**
APGAR (1 min)	8 (8–8)	7 (5.5–8)	**0.0005**
APGAR (5 min)	9 (9–9)	9 (7–9)	**0.0188**
Weight (g)	2750 (2628–3273)	1935 (1650–2773)	**0.002**
Length (cm)	50 (49–50)	44 (42–48.3)	**<0.0001**
Cephalic Perimeter (cm)	35 (34–35)	31 (29.4–34)	**<0.0001**
Temperature (°C)	36.7 (36.6–36.8)	36.4 (36–36.9)	0.1865
Cardiac Rate (bpm)	140 (135.8–144.8)	152 (145–160)	**0.003**
Respiratory Rate (bpm)	45.3 (3.7)	57 (9.9)	**0.0002**
SaO_2_ (%)	98 (97–98.8)	96.5 (94–99)	0.1651
FiO_2_ (%)	21 (21–21)	35 (30–100)	**<0.0001**
Hemoglobin (g/dL)	NA	16.9 +/− 2.1	NA
Ht (%)	NA	50.4 +/− 7	NA
Leukocytes (×10^9^/L)	NA	10,550 (7450–14,050)	NA
Lymphocytes (×10^9^/L)	NA	3145 (2158–5148)	NA
Monocytes (×10^9^/L)	NA	899 +/− 482.6	NA
Platelets	NA	212,586 +/− 56,030	NA
Neutrophils (×10^9^/L)	NA	6030 +/− 3008	NA
Glucose (mg/dL)	NA	73.7 (59.2–100.1)	NA
Creatinine (mg/dL)	NA	0.7 (0.6–0.8)	NA
Urea (mg/dL)	NA	16.4 (11.6–21.6)	NA

* Significant values are highlighted in bold.

## Data Availability

All the data are available in the manuscript and Supplementary Materials.

## Supplementary Materials

The following supporting information can be downloaded at: https://www.mdpi.com/article/10.3390/metabo14010041/s1, Table S1: Compound classification; Table S2: Urinary concentration values for NICU newborns and reference values found in HMDB (www.hmdb.ca, accessed on 30 May 2023) and Pubmed (https://pubmed.ncbi.nlm.nih.gov, accessed on 30 May 2023) [6,11,12,13,14,15,16,17,18,19,20,21,22,23,24,25,26,27,28,29,30,31,32,33,34,35,36,37,38,39,40,41,42,43,44,45,46,47,48,49,50,51,52]; Table S3: Perinatal characteristics and maternal history of newborns diagnosed with BPD and non-BPD newborns; Table S4: Fold Changes (FC) for Healthy vs. NICU newborns. Table S5: Pathway analysis: healthy vs. NICU newborns; Figure S1: Volcano plot comparison of BPD newborns (cesarean vs. va, c/v).
