# Supplementary material for: The Urinary Metabolome of Newborns with Perinatal Complications
Source: Metabolites. 2024 Jan 10;14(1):41. doi: 10.3390/metabo14010041 (PMC10819924; doi:10.3390/metabo14010041)
Supplement: Supplementary file 1 [file metabolites-14-00041-s001.zip › Supplementary Table S1.pdf]

Supplementary Table S1: Compound classification. For classification into compound classes, ClassyFire (http://classyfire.wishartlab.com) was used.

HMDB	Class
HMDB0000043	Amino acids, peptides, and analogues
HMDB0000056	Amino acids, peptides, and analogues
HMDB0000064	Amino acids, peptides, and analogues
HMDB0000070	Amino acids, peptides, and analogues
HMDB0000092	Amino acids, peptides, and analogues
HMDB0000099	Amino acids, peptides, and analogues
HMDB0000112	Amino acids, peptides, and analogues
HMDB0000123	Amino acids, peptides, and analogues
HMDB0000128	Amino acids, peptides, and analogues
HMDB0000148	Amino acids, peptides, and analogues
HMDB0000158	Amino acids, peptides, and analogues
HMDB0000159	Amino acids, peptides, and analogues
HMDB0000161	Amino acids, peptides, and analogues
HMDB0000162	Amino acids, peptides, and analogues
HMDB0000167	Amino acids, peptides, and analogues
HMDB0000168	Amino acids, peptides, and analogues
HMDB0000172	Amino acids, peptides, and analogues
HMDB0000177	Amino acids, peptides, and analogues
HMDB0000181	Amino acids, peptides, and analogues
HMDB0000182	Amino acids, peptides, and analogues
HMDB0000187	Amino acids, peptides, and analogues
HMDB0000191	Amino acids, peptides, and analogues
HMDB0000206	Amino acids, peptides, and analogues
HMDB0000214	Amino acids, peptides, and analogues
HMDB0000267	Amino acids, peptides, and analogues
HMDB0000271	Amino acids, peptides, and analogues
HMDB0000446	Amino acids, peptides, and analogues
HMDB0000450	Amino acids, peptides, and analogues
HMDB0000452	Amino acids, peptides, and analogues
HMDB0000479	Amino acids, peptides, and analogues
HMDB0000517	Amino acids, peptides, and analogues
HMDB0000532	Amino acids, peptides, and analogues
HMDB0000562	Amino acids, peptides, and analogues
HMDB0000641	Amino acids, peptides, and analogues
HMDB0000650	Amino acids, peptides, and analogues

HMDB0000679	Amino acids, peptides, and analogues
HMDB0000687	Amino acids, peptides, and analogues
HMDB0000696	Amino acids, peptides, and analogues
HMDB0000725	Amino acids, peptides, and analogues
HMDB0000766	Amino acids, peptides, and analogues
HMDB0000812	Amino acids, peptides, and analogues
HMDB0000866	Amino acids, peptides, and analogues
HMDB0000883	Amino acids, peptides, and analogues
HMDB0000904	Amino acids, peptides, and analogues
HMDB0000959	Amino acids, peptides, and analogues
HMDB0001138	Amino acids, peptides, and analogues
HMDB0001539	Amino acids, peptides, and analogues
HMDB0001904	Amino acids, peptides, and analogues
HMDB0002005	Amino acids, peptides, and analogues
HMDB0002931	Amino acids, peptides, and analogues
HMDB0003148	Amino acids, peptides, and analogues
HMDB0003334	Amino acids, peptides, and analogues
HMDB0003357	Amino acids, peptides, and analogues
HMDB0003911	Amino acids, peptides, and analogues
HMDB0004620	Amino acids, peptides, and analogues
HMDB0006028	Amino acids, peptides, and analogues
HMDB0006029	Amino acids, peptides, and analogues
HMDB0006344	Amino acids, peptides, and analogues
HMDB0011745	Amino acids, peptides, and analogues
HMDB0011756	Amino acids, peptides, and analogues
HMDB0011757	Amino acids, peptides, and analogues
HMDB0013713	Amino acids, peptides, and analogues
HMDB0032055	Amino acids, peptides, and analogues
HMDB0061684	Amino acids, peptides, and analogues
HMDB0094701	Amino acids, peptides, and analogues
HMDB0240251	Amino acids, peptides, and analogues
HMDB0255052	Amino acids, peptides, and analogues
HMDB0302754	Amino acids, peptides, and analogues
HMDB0000462	Azoles
HMDB0000500	Benzene and substituted derivatives
HMDB0000714	Benzene and substituted derivatives
HMDB0001870	Benzene and substituted derivatives
HMDB0001895	Benzene and substituted derivatives
HMDB0013678	Benzene and substituted derivatives

HMDB0000306	Benzene and substituted derivatives
HMDB0012275	Benzene and substituted derivatives
HMDB0000669	Benzene and substituted derivatives
HMDB0000707	Benzene and substituted derivatives
HMDB0000209	Benzene and substituted derivatives
HMDB0001276	Carboximidic acids and derivatives
HMDB0002064	Carboximidic acids and derivatives
HMDB0002172	Carboximidic acids and derivatives
HMDB0000237	Carboxylic acids and derivatives
HMDB0001873	Carboxylic acids and derivatives
HMDB0000134	Carboxylic acids and derivatives
HMDB0000176	Carboxylic acids and derivatives
HMDB0000202	Carboxylic acids and derivatives
HMDB0000254	Carboxylic acids and derivatives
HMDB0000661	Carboxylic acids and derivatives
HMDB0000691	Carboxylic acids and derivatives
HMDB0000072	Carboxylic acids and derivatives
HMDB0000094	Carboxylic acids and derivatives
HMDB0000193	Carboxylic acids and derivatives
HMDB0013327	Carboxylic acids and derivatives
HMDB0001964	Cinnamic acids and derivatives
HMDB0000226	Diazines
HMDB0000262	Diazines
HMDB0000300	Diazines
HMDB0000630	Diazines
HMDB0000201	Fatty Acyls
HMDB0000222	Fatty Acyls
HMDB0000552	Fatty Acyls
HMDB0000651	Fatty Acyls
HMDB0000756	Fatty Acyls
HMDB0000791	Fatty Acyls
HMDB0000824	Fatty Acyls
HMDB0000848	Fatty Acyls
HMDB0002013	Fatty Acyls
HMDB0002014	Fatty Acyls
HMDB0002250	Fatty Acyls
HMDB0002366	Fatty Acyls
HMDB0005066	Fatty Acyls
HMDB0006320	Fatty Acyls

HMDB0006464	Fatty Acyls
HMDB0006469	Fatty Acyls
HMDB0013124	Fatty Acyls
HMDB0013125	Fatty Acyls
HMDB0013126	Fatty Acyls
HMDB0013127	Fatty Acyls
HMDB0013128	Fatty Acyls
HMDB0013129	Fatty Acyls
HMDB0013130	Fatty Acyls
HMDB0013161	Fatty Acyls
HMDB0013205	Fatty Acyls
HMDB0013207	Fatty Acyls
HMDB0013326	Fatty Acyls
HMDB0013328	Fatty Acyls
HMDB0013330	Fatty Acyls
HMDB0013331	Fatty Acyls
HMDB0013332	Fatty Acyls
HMDB0013333	Fatty Acyls
HMDB0013334	Fatty Acyls
HMDB0013335	Fatty Acyls
HMDB0013336	Fatty Acyls
HMDB0013340	Fatty Acyls
HMDB0241094	Fatty Acyls
HMDB0241102	Fatty Acyls
HMDB0000039	Fatty Acyls
HMDB0000317	Fatty Acyls
HMDB0000407	Fatty Acyls
HMDB0000482	Fatty Acyls
HMDB0000535	Fatty Acyls
HMDB0000555	Fatty Acyls
HMDB0000622	Fatty Acyls
HMDB0000718	Fatty Acyls
HMDB0000754	Fatty Acyls
HMDB0000892	Fatty Acyls
HMDB0001987	Fatty Acyls
HMDB0061112	Fatty Acyls
HMDB0005876	Fatty Acyls
HMDB0004812	Furans
HMDB0002815	Glycerophospholipids

HMDB0007874	Glycerophospholipids
HMDB0007886	Glycerophospholipids
HMDB0007892	Glycerophospholipids
HMDB0007893	Glycerophospholipids
HMDB0007977	Glycerophospholipids
HMDB0007985	Glycerophospholipids
HMDB0007991	Glycerophospholipids
HMDB0007993	Glycerophospholipids
HMDB0008002	Glycerophospholipids
HMDB0008052	Glycerophospholipids
HMDB0008057	Glycerophospholipids
HMDB0008083	Glycerophospholipids
HMDB0008089	Glycerophospholipids
HMDB0008276	Glycerophospholipids
HMDB0008688	Glycerophospholipids
HMDB0008690	Glycerophospholipids
HMDB0010379	Glycerophospholipids
HMDB0010382	Glycerophospholipids
HMDB0010383	Glycerophospholipids
HMDB0010384	Glycerophospholipids
HMDB0010385	Glycerophospholipids
HMDB0010386	Glycerophospholipids
HMDB0010393	Glycerophospholipids
HMDB0010394	Glycerophospholipids
HMDB0010395	Glycerophospholipids
HMDB0010396	Glycerophospholipids
HMDB0010405	Glycerophospholipids
HMDB0011128	Glycerophospholipids
HMDB0012108	Glycerophospholipids
HMDB0013406	Glycerophospholipids
HMDB0013417	Glycerophospholipids
HMDB0013422	Glycerophospholipids
HMDB0029205	Glycerophospholipids
HMDB0029206	Glycerophospholipids
HMDB0029220	Glycerophospholipids
HMDB0029221	Glycerophospholipids
HMDB0000008	Hydroxy acids and derivatives
HMDB0000190	Hydroxy acids and derivatives
HMDB0000729	Hydroxy acids and derivatives

HMDB0000011	Hydroxy acids and derivatives
HMDB0000156	Hydroxy acids and derivatives
HMDB0000336	Hydroxy acids and derivatives
HMDB0000337	Hydroxy acids and derivatives
HMDB0059655	Hydroxy acids and derivatives
HMDB0000034	Imidazopyrimidines
HMDB0000132	Imidazopyrimidines
HMDB0000157	Imidazopyrimidines
HMDB0000289	Imidazopyrimidines
HMDB0000292	Imidazopyrimidines
HMDB0000897	Imidazopyrimidines
HMDB0003320	Indoles and derivatives
HMDB0000738	Indoles and derivatives
HMDB0029739	Indoles and derivatives
HMDB0000197	Indoles and derivatives
HMDB0000671	Indoles and derivatives
HMDB0000763	Indoles and derivatives
HMDB0000929	Indoles and derivatives
HMDB0002302	Indoles and derivatives
HMDB0000259	Indoles and derivatives
HMDB0000303	Indoles and derivatives
HMDB0004095	Indoles and derivatives
HMDB0000243	Keto acids and derivatives
HMDB0000208	Keto acids and derivatives
HMDB0000225	Keto acids and derivatives
HMDB0000019	Keto acids and derivatives
HMDB0000060	Keto acids and derivatives
HMDB0000695	Keto acids and derivatives
HMDB0000251	Organic sulfonic acids and derivatives
HMDB0000682	Organic sulfuric acids and derivatives
HMDB0011635	Organic sulfuric acids and derivatives
HMDB0062551	Organic sulfuric acids and derivatives
HMDB0000002	Organonitrogen compounds
HMDB0000087	Organonitrogen compounds
HMDB0000149	Organonitrogen compounds
HMDB0000164	Organonitrogen compounds
HMDB0000870	Organonitrogen compounds
HMDB0000906	Organonitrogen compounds
HMDB0001256	Organonitrogen compounds

HMDB0001257	Organonitrogen compounds
HMDB0001414	Organonitrogen compounds
HMDB0000925	Organonitrogen compounds
HMDB0001432	Organonitrogen compounds
HMDB0013222	Organonitrogen compounds
HMDB0000062	Organonitrogen compounds
HMDB0000097	Organonitrogen compounds
HMDB0003070	Organooxygen compounds
HMDB0000122	Organooxygen compounds
HMDB0000139	Organooxygen compounds
HMDB0000943	Organooxygen compounds
HMDB0000956	Organooxygen compounds
HMDB0010319	Organooxygen compounds
HMDB0253456	Organooxygen compounds
HMDB0000684	Organooxygen compounds
HMDB0000033	Peptidomimetics
HMDB0000020	Phenols
HMDB0000440	Phenols
HMDB0000068	Phenols
HMDB0000073	Phenols
HMDB0000216	Phenols
HMDB0000022	Phenols
HMDB0000118	Phenols
HMDB0000071	Purine nucleosides
HMDB0000085	Purine nucleosides
HMDB0000101	Purine nucleosides
HMDB0000050	Purine nucleosides
HMDB0000133	Purine nucleosides
HMDB0000195	Purine nucleosides
HMDB0000299	Purine nucleosides
HMDB0000058	Purine nucleotides
HMDB0000229	Pyridine nucleotides
HMDB0000232	Pyridines and derivatives
HMDB0000699	Pyridines and derivatives
HMDB0004193	Pyridines and derivatives
HMDB0000012	Pyrimidine nucleosides
HMDB0000014	Pyrimidine nucleosides
HMDB0000273	Pyrimidine nucleosides
HMDB0000296	Pyrimidine nucleosides

HMDB0000884	Pyrimidine nucleosides
HMDB0000715	Quinolines and derivatives
HMDB0000842	Quinolines and derivatives
HMDB0257047	Quinolines and derivatives
HMDB0001348	Sphingolipids
HMDB0010169	Sphingolipids
HMDB0012100	Sphingolipids
HMDB0012101	Sphingolipids
HMDB0013462	Sphingolipids
HMDB0013463	Sphingolipids
HMDB0013465	Sphingolipids
HMDB0013466	Sphingolipids
HMDB0013467	Sphingolipids
HMDB0013469	Sphingolipids
HMDB0240613	Sphingolipids
