# Supplementary material for: The Urinary Metabolome of Newborns with Perinatal Complications
Source: Metabolites. 2024 Jan 10;14(1):41. doi: 10.3390/metabo14010041 (PMC10819924; doi:10.3390/metabo14010041)
Supplement: Supplementary file 1 [file metabolites-14-00041-s001.zip › Supplementary Table S2.pdf]

Supplementary Table S2: Urinary concentration values for NICU newborns and reference values found in HMDB (www.hmdb.ca, accessed on May 30, 2023) and Pubmed (https://pubmed.ncbi.nlm.nih.gov, accessed on May 30, 2023 )

| Metabolite | HMDB CODE | This study<br><br>µmol/mmol Cr | Normal values (HMDB)<br>µmol/mmol Cr | Abnormal values (HMDB)<br>µmol/mmol Cr | Values reported in other studies searched in Pubmed |
|---|---|---|---|---|---|
| 1-Methylnicotinamide | HMDB0000699 | 61.37 (40.64-92.98) | 0.82 (0.26-2.3)[11] | - | |
| 3-Methoxytyramine | HMDB0000022 | 0.09 (0.07-0.14) | - | - | |
| 5-Hydroxylysine | HMDB0000450 | 5.91 (4.13-8.16) | 4.97 - 116.9[12] | - | 37(13-114) µM/g Cr[13] |
| 7-Methylguanine | HMDB0000897 | 10.79 +/- 0.86 | - | - | |
| $\alpha$-Aminobutyric acid | HMDB0000650, HMDB0000452 | 0.48 (0.14-1.22) | 1.13 +/- 4.52[14] | - | 15.2(8-26) µM/g Cr[13] |
| Asymmetric dimethylarginine | HMDB0001539 | 5.76 (4.47-7.58) | 0.98-4.87[6] | - | 14.8 (10.3–21.7) mmol/mmol Cr [15]<br>12.2 +/- 4.6[16] |
| Alanine | HMDB0000161 | 75.45 (53.05-122.77) | 32.25-147.8[6] | 427.6-681.4[17] | 1161(630-2227) µM/g Cr [13] |
| Allantoin | HMDB0000462 | 16.48 (12.33-22.54) | - | - | 19.1 +/-18.5[18] |
| Arginine | HMDB0000517 | 13.50 (9.47-21.18) | 6.82-30.52[6] | - | 424(288-731) µM/g Cr[13] |
| Asparagine | HMDB0000168 | 12.77 (7.96-24.28) | 0.42-10.5[6] | 20.7-447[19] | 528(264-1033) µM/g Cr[13] |
| Aspartic acid | HMDB0000191 | 14.27 (9.19-21.64) | 5.57-34.61[6] | - | 2.6(0-16.5) µM/g Cr[13] |
| $\beta$-Alanine | HMDB0000056 | 7.24 (5.07-11.33) | 10.18 +/- 10.18[14] | - | 1154(627-2224) µM/g Cr[13] |
| Betaine | HMDB0000043 | 398.69 (286.91-503.31) | 74.09-410.3[6] | - | 13.62 ± 3.71[20] |

| | | | | | |
|---|---|---|---|---|---|
| Carnosine | HMDB0000033 | 21.55 (8.70-83.05) | 0.47-35.49[6] | - | 1.45 ± 0.61µmol/mmol Cr [20]<br>84(29-269) µM/g Cr[13] |
| Choline | HMDB0000097 | 8.08 (5.14-11.09) | 3.99-50.7[6] | - | |
| Citrulline | HMDB0000904 | 1.81 (1.26-3.21) | 0 - 10.97[12] | 63.8-549.4[19] | 33(14-73) µM/g Cr[13] |
| Creatine | HMDB0000064 | 21.86 (7.18-123.08) | 4.66-455.3[6] | - | |
| Cystathionine | HMDB0000099 | 10.73 (6.39-17.60) | 32.79 +/- 27.13[14] | - | 349(153-703) µM/g Cr[13] |
| Cytidine | HMDB0000630 | 0.27 (0.21-0.42) | 1.1-10.7[21] | - | |
| Diacetylspermine | HMDB0002172 | 2.29 (1.16-3.10) | 0.37-3.11[6] | - | |
| Dimethylamine | HMDB0000087 | 71.95 (44.20-116.10) | 20-60[21] | - | 282 +/- 44 µmol/mmol Cr [16] |
| Dopamine | HMDB0000073 | 0.29 +/- 0.02 | 0.13-2.63[6] | 0.49 +/- 0.06[22] | |
| Ethanolamine | HMDB0000149 | 296.05 +/- 25.65 | 31.89-427.74 [6] | 204-1600[23] | 3243(1345-5142) µM/g Cr[13] |
| Glutamine | HMDB0000641 | 64.71 (51.53-108.46) | 16.16-134.4[6] | 0-8[24] | 1144(647-2152) µM/g Cr[13] |
| Glutamic acid | HMDB0000148 | 6.31 (4.56-16.08) | 1.96-35.26[6] | - | 96(48-178) µM/g Cr[13] |
| Glycine | HMDB0000123 | 419.12 (254.10-781.58) | 102.6-853.6[6] | - | 7408(3343-14352) µM/g Cr[13] |
| Guanine | HMDB0000132 | 0.28 (0.17-0.41) | - | - | |
| Histidine | HMDB0000177 | 63.73 (36.37-98.87) | 21.09-188.1[6] | 246.7-642.7[19] | 1601(858-3265) µM/g Cr[13] |

| | | | | | |
|---|---|---|---|---|---|
| Histamine | HMDB0000870 | 0.012 +/- 0.001 | 0.01-0.04[6] | - | |
| Homocitrulline | HMDB0000679 | 3.14 (2.45-4.68) | 0 - 10.18[12] | - | |
| Hypoxanthine | HMDB0000157 | 17.09 (10.51-28.19) | <5.2[25] | 88[26] | 17 +/- 38 μM/μM Cr[18] |
| Isoleucine | HMDB0000172 | 2.35 (1.32-4.29) | 0.29-7.30[6] | - | |
| Inosine | HMDB0000195 | 0.59 (0.38-1.00) | 0-25[21] | - | |
| Kynurenine | HMDB0000684 | 0.18 (0.08-0.70) | 0.02-0.71[6] | - | |
| Leucine | HMDB0000687 | 6.47 (4.23-15.05) | 0.33-13.41[6] | 53.9-17.7[19] | |
| Lysine | HMDB0000182 | 119.52 (38.07-209.52) | 10.5-140.5[6] | 268.3-365.2[19] | 2878(1304-7527) μM/g Cr[13] |
| Methionine | HMDB0000696 | 3.68 (2.76-5.71) | 0.54-4.81[6] | - | 34(14-69) μM/g Cr[13] |
| Methionine Sulfoxide | HMDB0002005 | 0.52 (0.21-0.99) | 0.6-3.59[6] | - | |
| Methylhistidine | HMDB0000479 | 42.71 (31.68-56.08) | 32.79 +/- 20.35[14] | - | 197(141-277) μM/g Cr[13] |
| N1-Acetylspermidine | HMDB0001276 | 2.06 (1.74-3.53) | - | - | |
| N-Acetylputrescine | HMDB0002064 | 20.14 (11.77-28.46) | - | - | |
| Nudifloramide | HMDB0004193 | 17.23 (12.63-22.96) | 0.71 (0.13-2.88)[11] | - | |

| | | | | | |
|---|---|---|---|---|---|
| Ornithine | HMDB0000214 | 7.11 (2.36-19.71) | 1.44-24.36[6] | 92.57-123.8[19] | 259(84-1051) µM/g Cr[13] |
| Phenylalanine | HMDB0000159 | 9.18 (7.09-15.78) | 4.14-14.15[6] | 414.7[27] | 122(74-207) µM/g Cr[13] |
| Proline | HMDB0000162 | 47.98 (34.13-109.22) | 9.5-78.2[6] | - | 1145(334-3459) µM/g Cr[13] |
| Putrescine | HMDB0001414 | 0.29 (0.17-0.46) | 0.03-3.42[6] | - | |
| Sarcosine | HMDB0000271 | 3.69 (2.01-5.49) | 0.55-9.98[6] | 2703 [28] | 192(113-514) µM/g Cr[13] |
| Serine | HMDB0000187 | 106.75 (74.87-156.83) | 32.19-145.5[6] | 341.6-735[19] | 1640(805-3327) µM/g Cr[13] |
| Serotonin | HMDB0000259 | 0.12 (0.10-0.15) | 0.1-0.35[6] | 0.22[29] | |
| Taurine | HMDB0000251 | 1107.39 (537.18-1691.0) | 74.64-284[6] | 1261[26] | 2939(289-10577) µM/g Cr[13] |
| Threonine | HMDB0000167 | 56.31 (24.51-119.66) | 9.24-96.17[6] | 226.2-1060[19] | 833(354-1810) µM/g Cr[13] |
| Trimethylamine N-oxide | HMDB0000925 | 5.62 (0.36-10.44) | 0.30-43.08[6] | - | |
| Total Dimethylarginine | HMDB0003334 + HMDB0001539 | 30.46 (22.86-37.28) | 5.33-18.11[6]<br><br>0.98-4.87[6] | - | 31.0 (21.7– 65.9) mmol/mmol Cr 26.1 +/- 4.5[16] |
| trans-4-Hydroxyproline | HMDB0000725 | 67.94 (43.53-132.88) | 8.12-92.19[6] | 1957[30] | 744(304-1397) µM/g Cr[13] |
| Tryptophan | HMDB0000929 | 3.82 (2.59-6.49) | 1.86-6.03[6] | - | 50(27-99) µM/g Cr[13] |
| Tryptamine | HMDB0000303 | 0.06 (0.04-0.08) | - | - | |
| Tyrosine | HMDB0000158 | 5.03 (2.60-7.72) | 2.1-14.81[6] | 35.15-369[19] | 112(62-238) µM/g Cr[13] |

| | | | | | |
|---|---|---|---|---|---|
| Tyramine | HMDB0000306 | 0.38 (0.27-0.52) | 0.30-2.54[6] | - | |
| Uracil | HMDB0000300 | 3.05 (1.76-4.24) | 8-110[21] | - | |
| Uridine | HMDB0000296 | 1.35 (0.71-2.90) | 0-15[21] | - | |
| Valine | HMDB0000883 | 8.60 (5.47-18.58) | 0.99-18.44[6] | - | 135(67-284) μM/g Cr[13] |
| Glucose | HMDB0000122 | 683.75 (382.16-1119.80) | 106.3-1193[6] | - | 30.67 ± 4.81μmol/mmol Cr [20] |
| C0 | HMDB0000062 | 2.87 (1.76-6.00) | 0.79-5.66[6] | - | |
| C2 | HMDB0000201 | 0.67 (0.40-1.58) | 0.38-1.86[6] | - | |
| C4:1 | HMDB0013126 | 0.02 (0.01-0.03) | 0.01-0.04[6] | - | |
| C4 | HMDB0002013 | 0.21 (0.10-0.37) | 0.05-0.33[6] | - | |
| C3OH | HMDB0013125 | 0.02 (0.01-0.05) | 0.01-0.06[6] | - | |
| C5:1 | HMDB0002366 | 0.07 (0.05-0.10) | 0.03-0.16[6] | - | |
| C5 | HMDB0013128 | 0.10 (0.05-0.25) | 0.02-0.29[6] | - | |
| C4OH | HMDB0013127 | 0.05 (0.03-0.09) | - | - | |
| C6:1 | HMDB0013161 | 0.04 (0.03-0.06) | 0.01-0.02[6] | - | |

| | | | | | |
|---|---|---|---|---|---|
| C5OH | HMDB0013132 | 0.14 (0.10-0.21) | 0.04-0.29[6] | - | |
| C5:1DC | HMDB0013129 | 0.04 (0.02-0.05) | 0.0-0.03[6] | - | |
| C5DC | HMDB0013130 | 0.06 (0.05-0.08) | 0.02-0.1[6] | - | |
| C8 | HMDB0000791 | 0.05 (0.04-0.09) | 0.02-0.31[6] | - | |
| C5MDC | HMDB0000552 | 0.08 (0.06-0.10) | 0.03-0.13[6] | - | |
| C9 | HMDB0006320 | 0.07 (0.06-0.09) | - | - | |
| C7DC | HMDB0013328 | 0.04 (0.03-0.05) | 0.02-0.09[6] | - | |
| C10:1 | HMDB0013205 | 0.08 (0.02-0.15) | 0.03-0.2[6] | - | |
| C12:1 | HMDB0013326 | 0.06 (0.02-0.08) | 0.02-0.14[6] | - | |
| C12DC | HMDB0013327 | 0.02 (0.01-0.03) | 0.01-0.29[6] | - | |
| C16:1 | HMDB0013207 | 0.03 (0.02-0.05) | 0.0-0.03[6] | - | |
| 2-Hydroxy-3-methylvaleric acid | HMDB0000317 | 0.04 (0.02-0.06) | - | - | |
| 2-Hydroxybutyric acid | HMDB0000008 | 2.53 (1.56-6.08) | 5.0 (0.0-10.0)[11] | - | |
| 2-hydroxyglutaric acid | HMDB0059655 | 18.25+/- 1.50 | 51.9 (13.7-97.3)[31] | 62[32] | 21.1 +/-5.5 µmol/mmol Cr [33] |

| | | | | | |
|---|---|---|---|---|---|
| 2-Hydroxyisobutyric acid | HMDB0000729 | 0.60 (0.35-0.97) | 0.1 (0.1-3.8)[31] | - | |
| 2-oxoadipic acid | HMDB0000225 | 12.41 (6.29-19.87) | - | 135[34] | |
| 2-oxoisocaproic acid | HMDB0000695 | 0.58 (0.39-0.87) | 0-0.2[21] | - | |
| 3,4-Dihydroxybutyric acid | HMDB0000337 | 5.26 (4.24-6.80) | 73.0 +/- 24.0 [11] | - | |
| 3-Deoxyglucosone | HMDB0005876 | 7.12 (5.01-8.52) | - | - | |
| 3-Hydroxybutyric acid | HMDB0000011 | 1.62 (0.90-5.40) | 0.72-23.3[6] | 51.04-56.47[35] | 16.28 +/- 3.50[33] |
| 3-Hydroxyisobutyric acid | HMDB0000336 | 12.33 (5.09-23.56) | - | - | |
| 3-Hydroxyisovaleric acid | HMDB0000754 | 7.21 (4.65-11.74) | 12.2 (0.1-43.2)[31] | 1154.8 [36] | |
| 3-Hydroxyphenylacetic acid | HMDB0000440 | 0.63 +/- 0.04 | - | 0.67 +/- 2.9[37] | |
| 3-Methyladipic acid | HMDB0000555 | 0.48 +/- 0.04 | - | - | |
| 4-Hydroxyphenylacetic acid | HMDB0000020 | 7.96(4.70-14.16) | 24.2 (8.6-73.2)[31] | 428.0 +/- 164.0[38] | |
| 4-Hydroxyphenylpyruvic acid | HMDB0000707 | 150.31(60.04-507.20) | 4.6 (0.1-21.3)[31] | 24.00 +/- 30.5[37] | |
| 5-Hydroxyindoleacetic acid | HMDB0000763 | 8.69 +/- 0.38 | 11.0 (4.0-18.0)[39] | 0.9 +/- 0.0[39] | |
| 5-Oxoproline | HMDB0000267 | 22.99 (19.74-38.23) | 7.3 (0.1-29.3)[31] | 1980 – 6040[40] | |
| alpha-Ketoglutaric acid | HMDB0000208 | 162.32 (71.28-284.43) | 30.79-398.2[6] | 385.200-1367.300 [41] | |

| | | | | | |
|---|---|---|---|---|---|
| alpha-Ketoisovaleric acid | HMDB0000019 | 1.81 (0.52-5.32) | 0.16 +/- 0.48 [42] | - | |
| Argininic acid | HMDB0003148 | 8.21 (3.83-13.95) | - | - | |
| Benzoic acid | HMDB0001870 | 0.52 (0.30-0.85) | - | - | |
| Butyric acid + Isobutyric acid | HMDB0000039 + HMDB0001873 | 3.49 (1.36-5.61) | 0.06-0.86[6] | - | |
| Caproic acid | HMDB0000535 | 0.46 (0.14-0.75) | 0.91 +/- 1.05[42] | - | |
| Caprylic acid | HMDB0000482 | 0.15 (0.04-0.22) | 0.39 +/- 0.37[42] | - | |
| cis-Aconitic acid | HMDB0000072 | 45.72 +/- 2.82 | 67.9 (14.3-100.7)[31] | 6.4-293.5[17] | |
| Citric acid | HMDB0000094 | 379.57 (228.21-614.87) | 39.6-893[6] | 998[32] | |
| Dimethylglycine | HMDB0000092 | 15.04 (11.87-21.42) | 15-220[21] | - | |
| Ethylmalonic acid | HMDB0000622 | 4.14 (3.10-5.81) | 0.012 +/- 0.00[33] | 2.56-10.26[43] | |
| 2,5-Furandicarboxylic acid | HMDB0004812 | 1.39 (0.64-3.43) | 2.7 (0.1-9.1)[31] | - | |
| Fumaric acid | HMDB0000134 | 10.34 (6.40-21.12) | 2.09-37.66[6] | 17-124[44] | |
| Glutaric acid | HMDB0000661 | 1.51 (0.74-3.15) | 1.896 +/- 0.862 [33] | 3.3-44.0[45] | 7.623 +/-3.321μmol/mmol Cr [33] |
| Glyceric acid | HMDB0000139 | 3.18 (1.06-7.97) | 21.6 (0.5-91.1) [31] | - | |
| Guanidinopropionic acid | HMDB0013222 | 13.71 (6.38-69.84) | - | - | |
| Guanidoacetic acid | HMDB0000128 | 11.30 (7.28-20.30) | 93-190 [21] | - | |

| | | | | | |
|---|---|---|---|---|---|
| | | | | | |
| Hippuric acid | HMDB0000714 | 27.57 (16.64-44.00) | 1.51-213[6] | - | 189.79 ± 5.24 µmol/mmol Cr[20] |
| Indolelactic acid | HMDB0000671 | 0.11 (0.07-0.22) | - | - | |
| Indoxyl sulfate | HMDB0000682 | 2.57 (1.67-4.03) | - | - | |
| Isocitric acid | HMDB0000193 | 45.72 +/- 2.77 | 19.0 (5.0-34.0)[11] | - | |
| Kynurenic acid | HMDB0000715 | 4.94 (3.81-7.06) | - | - | |
| Lactic acid | HMDB0000190 | 165.77 (85.81-350.52) | 46.99-774.2[6] | 352[32] | |
| Maleic acid | HMDB0000176 | 1.14 (0.73-1.78) | 10.4 [31] | - | |
| Malic acid | HMDB0000156 | 64.79 (32.94-109.83) | 36.1(12.4-121.6[31] | 110 [32] | |
| Malonic acid | HMDB0000691 | 0.92 +/- 0.07 | - | 2170.7-5438.1 [46] | |
| Methylmalonic acid | HMDB0000202 | 0.87 (0.36-1.23) | 0.42-19.74[6] | - | 32.192+/-6.937µmol/mmol Cr [33] |
| Orotic acid | HMDB0000226 | 2.05 +/- 0.15 | 0.061 +/- 0.003[33] | >20 [47] | 1.129 +/- 0.874µmol/mmol Cr [33] |
| Phenylacetic acid | HMDB0000209 | 0.12 (0.08-0.19) | - | 55.6 +/- 87.3[37] | |
| Phenylacetylglutamine | HMDB0006344 | 22.67 (17.32-33.98) | - | - | |
| p-Hydroxyhippuric acid | HMDB0013678 | 4.55 (3.40-7.47) | 4.2-19.59[6] | - | |
| Pipecolic acid | HMDB0000070 | 0.15 (0.14-0.24) | 0 - 25.33 [12] | - | |

| | | | | | |
|---|---|---|---|---|---|
| Propionic acid | HMDB0000237 | 8.40 (3.04-13.01) | 0-76[21] | - | |
| Pyruvic acid | HMDB0000243 | 7.17 (5.60-13.41) | 9.55-89.06[6] | - | |
| Quinaldic acid | HMDB0000842 | 0.01 (0.01-0.02) | - | - | |
| Quinolinic acid | HMDB0000232 | 18.29 (14.14-24.02) | 0.67 +/- 1.34 [42] | - | |
| Shikimic acid | HMDB0003070 | 0.68 +/- 0.04 | - | - | |
| Succinic acid | HMDB0000254 | 111.02 (31.39-241.29) | 4.35-81.2[6] | 75-185 [44] | |
| Tartaric acid | HMDB0000956 | 1.47 (0.95-2.88) | 0-60[21] | - | |
| Threonic acid | HMDB0000943 | 89.49 (69.00-103.31) | 63.0 +/- 21.0 [11] | - | |
| Uric acid | HMDB0000289 | 927.36 +/- 78.40 | 218.8-4202[6] | 20 [26] | |
| Tiglylglycine | HMDB0000959 | 0.79 (0.55-0.97) | 0-3.8[48] | 87[49] | |
| Xanthine | HMDB0000292 | 12.90 (7.06-23.41) | <40 [50] | 991[26] | |
| Xanthosine | HMDB0000299 | 2.14 (1.63-3.42) | - | - | |
| N1-Acetyl-Lysine | HMDB0000446 | 0.67 (0.48-1.15) | - | - | |
| N6-Acetyl-Lysine | HMDB0000206 | 1.14 (0.90-1.49) | - | - | |
| N-Acetyl-Alanine | HMDB0000766 | 4.00 (1.75-8.27) | - | - | |

| | | | | | |
|---|---|---|---|---|---|
| N-Acetyl-Arginine | HMDB0004620 | 1.84 (1.07-2.66) | - | - | |
| N-Acetyl-Asparagine | HMDB0006028 | 2.69 (2.05-4.40) | - | - | |
| N-Acetyl-Aspartic acid | HMDB0000812 | 26.26 (18.78-40.70) | 33.2 (4.9-65.5) [31] | - | |
| N-Acetyl-Glutamine | HMDB0006029 | 4.12 +/- 0.22 | - | - | |
| N-Acetyl-Glutamic acid | HMDB0001138 | 3.47 (2.78-4.83) | 0-42[21] | - | |
| N-Acetyl-Glycine | HMDB0000532 | 4.16 (3.28-5.36) | 0 [51] | 170 +/- 18[51] | |
| N-Acetyl-Histidine | HMDB0032055 | 8.13 (3.72-9.89) | - | - | |
| N-Acetyl-Isoleucine | HMDB0061684 | 0.47 (0.32-0.74) | - | - | |
| N-Acetyl-Leucine | HMDB0011756 | 0.54 (0.34-1.12) | - | - | |
| N-Acetyl-Methionine | HMDB0011745 | 1.22 (0.91-1.40) | - | - | |
| N-Acetyl-Proline | HMDB0094701 | 0.43 (0.25-0.60) | - | - | |
| N-Acetyl-Serine | HMDB0002931 | 5.02 (2.63-8.14) | - | - | |
| N-Acetyl-Tyrosine | HMDB0000866 | 0.22 (0.1-0.55) | 1.6 (0-6.4) [52] | 75 (0-781)[52] | |
| N-Acetyl-Valine | HMDB0011757 | 0.73 (0.53-0.83) | - | - | |
