# Supplementary material for: The Urinary Metabolome of Newborns with Perinatal Complications
Source: Metabolites. 2024 Jan 10;14(1):41. doi: 10.3390/metabo14010041 (PMC10819924; doi:10.3390/metabo14010041)
Supplement: Supplementary file 1 [file metabolites-14-00041-s001.zip › Supplementary Table S3.pdf]

Supplementary Table S3: Perinatal characteristics and maternal history of newborns diagnosed with BPD and non-BPD newborns.

**Variable**	**BPD (N=12)**	**Non-BPD (N=26)**	**p-value***
**Perinatal outcomes**			
Male Gender, n (%)	9 (75)	20 (77)	0.89
Gestational Age (Wks), median (Q1-Q3)	32.4 (30-33.6)	36.4 (35.5-38.2)	**<0.0001**
Vaginal Birth, n (%)	5 (41)	15 (58)	0.35
Reanimation, n (%)	9 (75)	10 (38)	**0.03**
Apgar (1 min), median (Q1-Q3)	6 (5.3-7.8)	8 (5.5-8)	0.32
Apgar (5 min), median (Q1-Q3)	9 (7.3-9)	9 (7-9)	0.97
Weight (g), mean ±SD	1668 ± 156.1	2420 ± 672.6	**0.0005**
Size(cm), mean ±SD	41.9 ± 1.8	46.4 ± 3.6	**0.0003**
Cephalic Perimeter (cm), mean ±SD	29.3 ± 1.1	32.3 ± 2.7	**0.0011**
Hypothermia, n (%)	1 (8)	7 (27)	0.19
Temperature (°C), mean ±SD	36.4 ± 0.6	36.3 ± 0.9	0.85
Cardiac Frequency (bpm), mean ±SD	148.7 ± 16.6	151.2 ± 14.5	0.64
Respiratory Frequency (bpm), median (Q1-Q3)	59 (56-60)	55.5 (49.5-68)	0.91
FiO2%, median (Q1-Q3)	80 (30-100)	30 (30-100)	0.11
SaO2%, median (Q1-Q3)	94.5 (91-98.8)	97 (94.8-99)	0.27
RT-PCR SARS-CoV-2, n (%)	0 (0)	0 (0)	NA
Antigen SARS-CoV-2, n (%)	0 (0)	0 (0)	NA
IgM SARS-CoV-2, n (%)	0 (0)	3 (12)	0.22
IgG SARS-CoV-2, n (%)	5 (42)	3 (12)	**0.03**
Days at NICU, median (Q1-Q3)	40 (30.8-52.3)	17 (12.5-27.3)	**<0.0001**
Respiratory Distress Syndrome, n (%)	5 (42)	0 (0)	**0.0004**
Transient Tachypnea, n (%)	5 (42)	12 (46)	0.79
Intrauterine pneumonia, n (%)	10 (83)	7 (27)	**0.001**
Persistent Pulmonary Hypertension, n (%)	3 (25)	3 (12)	0.29
Pneumothorax, n (%)	0 (0)	2 (8)	0.32
Respiratory Hypoxemic Failure, n (%)	1 (8)	0 (0)	0.13
Early Onset Sepsis, n (%)	4 (33)	4 (15)	0.20
Necrotizing Enterocolitis 1 A, n (%)	1 (8)	1 (4)	0.56
Meconium Aspiration Syndrome, n (%)	0 (0)	3 (12)	0.22
Neonatal Depression by Anesthetics, n (%)	0 (0)	3 (12)	0.22
Hypoxic Ischemic Encephalopathy stage II with seizures, n (%)	0 (0)	3 (12)	0.22

Hypoxic Ischemic Encephalopathy stage II without seizures, n (%)	1 (8)	5 (19)	0.39
Intrauterine Growth Restriction, n (%)	0 (0)	3 (12)	0.22
Liver Hematoma, n (%)	1 (8)	1 (4)	0.56
Hypertrophic Cardiomyopathy, n (%)	0 (0)	0 (0)	NA
Patent Ductus Arteriosus, n (%)	4 (33)	1 (4)	**0.012**
Pharmacological Treatment, n (%)	3 (25)	0 (0)	**0.007**
Neonatal sepsis, n (%)	6 (50)	5 (19)	0.05
Antibiotics, n (%)	11 (92)	17 (65)	0.08
Nitric oxide, n (%)	0 (0)	1 (4)	0.49
Caffeine, n (%)	7 (58)	0 (0)	**<0.0001**
Surfactant, n (%)	5 (42)	0 (0)	**0.0004**
Lower Airway Infection, n (%)	0 (0)	1 (4)	0.49
Intubation, n (%)	9 (75)	9 (35)	**0.02**
Oxygen Exposure Time <1 month, n (%)	1 (8)	20 (77)	**<0.0001**
Oxygen Exposure Time >1 month, n (%)	11 (92)	6 (23)	**<0.0001**
Discharge with Oxygen, n (%)	12 (100)	9 (35)	**0.0002**
**Laboratory data**			
Hemoglobin (g/dL), mean ±SD	16.2 ± 2.7	17.2 ± 1.7	0.18
Hematocrit %, mean ±SD	48.2 ± 8.7	51.4 ± 5.9	0.19
Leukocytes ($x10^9$/L), mean ±SD	8383 ± 2447	12557 ± 4537	**0.005**
Lymphocytes ($x10^9$/L), median (Q1-Q3)	2775 (1795-4650)	3460 (2403-5373)	0.16
Monocytes ($x10^9$/L), mean ±SD	696.7 ± 418.9	992.4 ± 488.6	0.07
Platelets ($x10^3$), median (Q1-Q3)	218 (147-261)	199 (182-263)	0.91
Neutrophils ($x10^9$/L), mean ±SD	4450 ± 2283	6760 ± 3056	**0.02**
Glucose (mg/dl), median (Q1-Q3)	70.2 (53.7- 90)	78 (59.2-112.3)	0.26
Creatinine (mg/dl), mean ±SD	0.6275 ± 0.1012	0.7362 ± 0.1672	**0.04**
Urea (mg/dl), median (Q1-Q3)	14.6 (11.1-19.1)	18.2 (13.21-23.6)	0.29
Lactate, median (Q1-Q3)	3.4 (2-4.3)	3.5 (2.7-6.8)	0.26
**Maternal Antecedents**			
Preeclampsia, n (%)	0 (0)	5 (19)	0.10
Gestational Diabetes, n (%)	3 (25)	3 (12)	0.29
Type 1 Diabetes, n (%)	0 (0)	0 (0)	NA
Type 2 Diabetes, n (%)	0 (0)	0 (0)	NA
Obesity, n (%)	1 (8)	0 (0)	0.31
Drug Abuse, n (%)	0 (0)	3 (12)	0.22
Syphilis, n (%)	0 (0)	0 (0)	NA
Human Immunodeficiency (AIDS), n (%)	0 (0)	1 (4)	0.49

Condyloma, n (%)	0 (0)	0 (0)	NA
Kidney Diseases, n (%)	0 (0)	0 (0)	NA
Anemia, n (%)	0 (0)	2 (8)	0.32
Urinary Infection, n (%)	2 (17)	11 (42)	0.12
Premature Labor, n (%)	12 (100)	14 (54)	**0.004**
Placenta Previa, n (%)	1 (8)	3 (12)	0.76
Chronic Arterial Hypertension, n (%)	0 (0)	0 (0)	NA

*Significant values (p<0.05) are highlighted in bold
