# Supplementary material for: The Urinary Metabolome of Newborns with Perinatal Complications
Source: Metabolites. 2024 Jan 10;14(1):41. doi: 10.3390/metabo14010041 (PMC10819924; doi:10.3390/metabo14010041)
Supplement: Supplementary file 1 [file metabolites-14-00041-s001.zip › Supplementary Table S4.pdf]

Supplementary Table S4: Fold Changes (FC) for Healthy vs. NICU newborns

	FC	log2(FC)	raw.pval
3-Deoxyglucosone	10.106	3.3371	2.11E-15
Benzoic acid	6.5667	2.7152	1.87E-08
alpha-Ketoisovaleric acid	13.783	3.7849	7.70E-08
C12DC	0.35144	-1.5087	9.05E-06
4-Hydroxyphenylpyruvic acid	2.4877	1.3148	0.00010237
Methionine	1.9403	0.95627	0.0001653
2-oxoisocaproic acid	2.4898	1.316	0.00017806
alpha-Ketoglutaric acid	2.9487	1.5601	0.00027767
Xanthosine	0.57869	-0.78914	0.00042464
Total Dimethylarginine	0.65794	-0.60397	0.00060146
cis-Aconitic acid	0.56326	-0.82814	0.00061839
Shikimic acid	0.66051	-0.59836	0.00069908
5-Oxoproline	0.56991	-0.81119	0.00086532
Methionine Sulfoxide	0.41352	-1.274	0.00092927
C6:1	1.6951	0.76138	0.0012013
Threonic acid	0.65705	-0.60593	0.0018799
Isocitric acid	0.51971	-0.94423	0.0020342
Proline	1.937	0.95385	0.0020893
Beta Alanine	1.6684	0.73848	0.0021301
Pipecolic acid	0.63847	-0.64732	0.0021893
2-Hydroxyisobutyric acid	2.0775	1.0548	0.003599
Histamine	2.2331	1.159	0.0036334
Allantoin	0.64908	-0.62354	0.0039944
N-Acetyl-Glutamic acid	0.7605	-0.39498	0.0041138
Indoxyl sulfate	0.51593	-0.95477	0.0044945
C10	0.62529	-0.6774	0.0046452
Xanthine	0.41308	-1.2755	0.0050177
Hippuric acid	0.30993	-1.69	0.0051193
C16:1	1.9368	0.95366	0.0052316
Carnosine	5.4296	2.4409	0.005247
Phenylacetylglutamine	0.50854	-0.97557	0.0052629
3-Hydroxyisobutyric acid	2.3125	1.2094	0.0056993
Asymmetric dimethylarginine	0.72637	-0.46123	0.0060758
Sarcosine	1.5937	0.67237	0.0067383
C12	0.56257	-0.82989	0.0067558
SM C16:0	0.28144	-1.8291	0.0071411

trans-4-Hydroxyproline	1.3946	0.47984	0.0077776
tyrosine/phe	0.72788	-0.45822	0.0084522
3-Methyladipic acid	0.59232	-0.75555	0.0087131
Homocitrulline	0.63303	-0.65966	0.0087446
Quinaldic acid	2.1707	1.1182	0.008909
C5:1DC	1.5823	0.66198	0.0112
Succinic acid	0.5222	-0.93732	0.011743
Guanidoacetic acid	0.65664	-0.60683	0.011901
Glutaric acid	0.60137	-0.73367	0.012265
3-Methoxytyramine	0.65053	-0.62031	0.013048
N-Acetyl-Alanine	1.8407	0.88026	0.015675
N-Acetyl-Methionine	0.72119	-0.47155	0.021251
arginine/ornithine	0.45585	-1.1334	0.021567
C5	1.9732	0.98056	0.025093
Pyruvic acid	2.5629	1.3578	0.026151
C4	2.0181	1.013	0.027957
Lysine	1.9017	0.92727	0.031381
Tyramine	0.6563	-0.60758	0.031989
Diacetylspermine	1.7577	0.81367	0.033004
4-Hydroxyphenylacetic acid	0.60061	-0.73551	0.037313
Cystathionine	1.6042	0.68184	0.038076
C0	1.7265	0.78785	0.039794
Ethylmalonic acid	0.67403	-0.56911	0.044712
3-Hydroxyisovaleric acid	1.3817	0.46645	0.045623
N-Acetyl-Serine	1.415	0.50079	0.04864
Dimethylamine	0.73838	-0.43756	0.04907
