# Supplementary material for: The Urinary Metabolome of Newborns with Perinatal Complications
Source: Metabolites. 2024 Jan 10;14(1):41. doi: 10.3390/metabo14010041 (PMC10819924; doi:10.3390/metabo14010041)
Supplement: Supplementary file 1 [file metabolites-14-00041-s001.zip › Supplementary Table S5.pdf]

Supplementary Table S5: Pathway Analysis: Healthy vs. NICU newborns

	Total Cmpd	Hits	Raw p	Holm adjust	FDR	Impact
Pantothenate and CoA biosynthesis	20	5	7.26E-06	0.00028326	0.00028326	0.04762
Tyrosine metabolism	42	8	0.00025548	0.0097081	0.0029331	0.41897
Cysteine and methionine metabolism	33	4	0.00027853	0.010306	0.0029331	0.30484
Valine, leucine and isoleucine degradation	40	6	0.00036238	0.013046	0.0029331	0.04432
Valine, leucine and isoleucine biosynthesis	8	6	0.00037604	0.013161	0.0029331	0
Citrate cycle (TCA cycle)	20	8	0.00069093	0.023492	0.0043338	0.39695
Ubiquinone and other terpenoid-quinone biosynthesis	18	2	0.00077786	0.025669	0.0043338	0.07692
Arginine and proline metabolism	36	10	0.0011344	0.036301	0.0053267	0.63139
Phenylalanine, tyrosine and tryptophan biosynthesis	4	3	0.0012292	0.038106	0.0053267	1
Glycine, serine and threonine metabolism	33	12	0.0020501	0.061504	0.0079955	0.74668
Propanoate metabolism	22	4	0.0022661	0.065717	0.0080344	0
beta-Alanine metabolism	21	5	0.0027642	0.077398	0.0089837	0.45522
Histidine metabolism	16	6	0.0037496	0.10124	0.010517	0.49999
Butanoate metabolism	15	5	0.0041723	0.10848	0.010517	0
Arginine biosynthesis	14	9	0.0045983	0.11496	0.010517	0.48223
Lipoic acid metabolism	28	4	0.0046253	0.11496	0.010517	0.0017
Purine metabolism	70	6	0.0049053	0.11496	0.010517	0.03965
Glyoxylate and dicarboxylate metabolism	32	10	0.0050758	0.11496	0.010517	0.28308
Lysine degradation	30	4	0.0051236	0.11496	0.010517	0.1227

Alanine, aspartate and glutamate metabolism	28	11	0.017862	0.35724	0.034831	0.67148
Biotin metabolism	10	1	0.031381	0.59624	0.058279	0
Glutathione metabolism	28	5	0.036569	0.65825	0.064828	0.12267
Pyrimidine metabolism	39	5	0.040608	0.69034	0.068857	0.13943
Glycolysis / Gluconeogenesis	26	3	0.058748	0.93996	0.095465	0.09806
Pyruvate metabolism	23	4	0.10596	1	0.1653	0.21967
Glycerolipid metabolism	16	1	0.12688	1	0.18327	0.09346
Pentose phosphate pathway	23	1	0.12688	1	0.18327	0
Selenocompound metabolism	20	1	0.13717	1	0.19106	0
Phenylalanine metabolism	8	3	0.18364	1	0.24697	0.35714
Tryptophan metabolism	41	6	0.19951	1	0.25936	0.39596
Taurine and hypotaurine metabolism	8	1	0.3709	1	0.46661	0.42857
Nitrogen metabolism	6	2	0.3927	1	0.47861	0
Primary bile acid biosynthesis	46	2	0.48452	1	0.54071	0.01516
Porphyrin metabolism	31	2	0.50342	1	0.54071	0
Fatty acid biosynthesis	47	1	0.50542	1	0.54071	0
D-Amino acid metabolism	15	1	0.51298	1	0.54071	0
Sphingolipid metabolism	32	1	0.51298	1	0.54071	0
Nicotinate and nicotinamide metabolism	15	4	0.54867	1	0.56311	0.13816
Glycerophospholipid metabolism	36	2	0.9018	1	0.9018	0.03906
