# Supplementary figures and images for: The Urinary Metabolome of Newborns with Perinatal Complications
Source: Metabolites. 2024 Jan 10;14(1):41. doi: 10.3390/metabo14010041 (PMC10819924; doi:10.3390/metabo14010041)

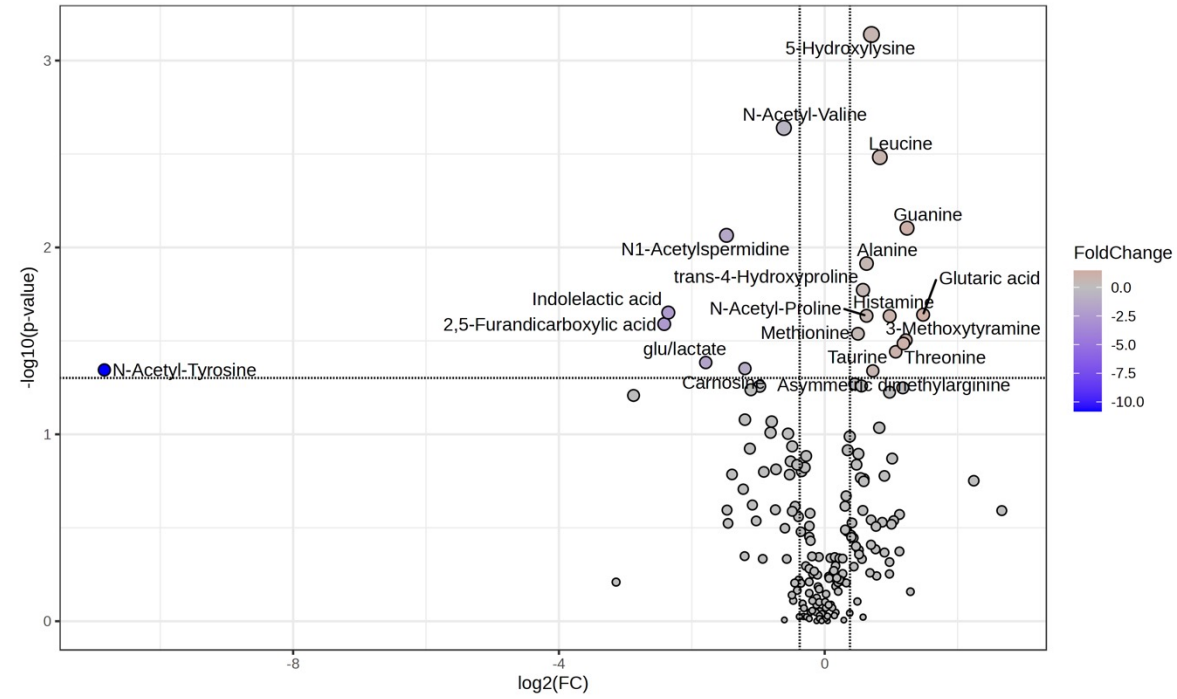

Supplementary Figure S1: Volcano plot: cesarean vs. vaginal delivery in BPD newborns (c/v)

Supplement: Supplementary file 1 [file metabolites-14-00041-s001.zip › Supplementary Figure S1.pdf]
